# Inhibition of P21-activated kinases 1 and 4 synergistically suppresses the growth of pancreatic cancer by stimulating anti-tumour immunity

**DOI:** 10.1186/s12964-024-01670-2

**Published:** 2024-05-27

**Authors:** Yi Ma, Chelsea Dumesny, Li Dong, Ching-Seng Ang, Khashayar Asadi, Yifan Zhan, Mehrdad Nikfarjam, Hong He

**Affiliations:** 1 Department of Surgery, Austin Precinct, University of Melbourne, Level 8, Lance Townsend Building, Austin Hospital, 145 Studley Road, Heidelberg, VIC Australia; 2 Department of Hepato-Pancreato-Biliary Surgery, Austin Health, Heidelberg, VIC Australia; 3 Mass Spectrometry and Proteomics Facility, Bio21 Molecular Science and Biotechnology Institute, University of Melbourne, Parkville, VIC Australia; 4 Department of Anatomical Pathology, Austin Health, Heidelberg, VIC Australia; 5 Department of General Surgery, Monash Health, Clayton, VIC Australia; 6 Drug Discovery, Shanghai Huaota Biopharm, Shanghai, China

**Keywords:** P21-activated kinases1&4, Intra-tumoral T cells, Pancreatic ductal adenocarcinoma

## Abstract

**Background:**

Pancreatic ductal adenocarcinoma (PDA) is one of the most lethal types of cancer, and *KRAS* oncogene occurs in over 90% of cases. P21-activated kinases (PAK), containing six members (PAK1 to 6), function downstream of KRAS. PAK1 and PAK4 play important roles in carcinogenesis, but their combinational effect remains unknown. In this study, we have determined the effect of dual inhibition of PAK1 and PAK4 in PDA progression using knockout (KO) cancer cell lines.

**Methods:**

Murine wild-type (WT) and PAK1KO pancreatic cancer cell lines were isolated from PAK1^+/+^ and PAK1^−/−^ KPC (*LSL-Kras*^*G12D/*+^*; LSL-Trp53 *^*R172H/*+^*; Pdx-1-Cre*) mice. KPC PAK4KO and KPC PAK1&4 KO cell lines were generated from KPC WT and KPC PAK1KO cell lines respectively using the CRISPR-CAS9 gene knockout technique. PAK WT and KO cell lines were used in mouse models of pancreatic tumours. Cells and tumour tissue were also used in flow cytometry and proteomic studies. A human PDA tissue microarray was stained by immunohistochemistry.

**Results:**

Double knock out of PAK1 and PAK4 caused complete regression of tumour in a syngeneic mouse model. PAK4KO inhibited tumour growth by stimulating a rapid increase of cytotoxic CD8+ T cell infiltration. PAK1KO synergistically with PAK4KO increased cytotoxic CD8+ T cell infiltration and stimulated a sustained infiltration of CD8+ T cells at a later phase to overcome the immune evasion in the PAK4KO tumour. The human PDA tissue microarray study showed the important role of PAK1 and PAK4 in intra-tumoral T-cell function.

**Conclusion:**

Our results demonstrated that dual inhibition of PAK1 and PAK4 synergistically suppressed PDA progression by stimulating cytotoxic CD8 + T cell response.

**Supplementary Information:**

The online version contains supplementary material available at 10.1186/s12964-024-01670-2.

## Background

Pancreatic ductal adenocarcinoma (PDA) is one of the most aggressive solid cancers, with a dismal five-year survival rate of 9.8% [1]. This is mainly due to late diagnosis and lack of effective treatment for advanced disease. While surgery can be curative, only 10–20% of patients have resectable tumours on diagnosis [2]. Despite advances in systemic therapy of solid malignancies, the development of targeted therapy in PDA remains stagnant. Gemcitabine-based chemotherapy and FOLFIRINOX are still the mainstay treatment of advanced PDA, but only extend overall survival on average, by two to three months [3, 4].

P21-activated kinase (PAK) has emerged as a potential therapeutic target. PAKs are a group of serine/threonine kinases that act downstream of KRAS, while KRAS mutations occur in over 90% of PDA [5–7]. There are six members of the PAK family, which are categorized into two groups: group 1 (PAK1 to 3) and group 2 (PAK4 to 6) [8]. Among the PAK proteins, PAK1 and PAK4 are mostly investigated for their role in tumorigenesis. In PDA, PAK1 was shown to inhibit cancer cell apoptosis, activate pancreatic stellate cells and down-regulate intra-tumoral CD4 + and CD8 + T cell infiltration [9–11]. PAK4 was reported to play a role in PDA cell apoptosis, cell cycle arrest and cancer cell stemness [12–14]. Recently, PAK4 was shown to suppress T cell response in melanoma, prostate cancer, and glioblastoma, which is consistent with an observed upregulation of intra-tumoral CD8 + T cells in PDA mouse model by PAK4 inhibitor PF-3758309 [10, 15–18].

Despite the pro-tumorigenic role of PAK1 and PAK4 in PDA, the development of clinically effective PAK1 or PAK4 inhibitors has not been successful. Given that both PAK1 and PAK4 play important roles in PDA biology, we hypothesize that the two may promote PDA tumour growth synergistically. Using PAK1 and PAK4 double knockout (KO) cell lines, we investigated their combinational effect in PDA which will guide future development of PAK-targeted therapy.

## Methods and methods

### Cell lines and cell culture

Murine WT and PAK1KO pancreatic cancer cell lines were isolated from PAK1^+/+^ and PAK1^−/−^ KPC mice as previously described [10]. KPC PAK4KO and KPC PAK1&4 KO cell lines were generated from KPC WT and KPC PAK1KO cell lines respectively using CRISPR-CAS technique. Cancer cells were cultured in Dulbecco’s Modified Eagle’s Medium (DMEM) supplemented with 5% fetal bovine serum (FBS) (Hyclone Laboratories, Melbourne, Australia) in a 37 °C incubator under humidified 5% CO_2_.

### Animal studies

All mouse studies were approved by the Austin Health Animal Ethics Committee (A2022/5797). Experimental C57BL6 and SCID mice were housed in the Austin Health Bioresource Facility and monitored for health criteria. For C57BL6 syngeneic mouse model, KPC WT, PAK1KO, PAK4KO and PAK1& 4 KO cells (0.5–1 × 10^6^ cells/100ul/mouse) were injected subcutaneously to the flank of 7 weeks old male C57BL6 mice. The mice were observed for one week or 4–6 weeks. Tumour growths were monitored using a calliper. Tumour volume (mm^3^) was calculated using the simplified ellipsoid formula:

$$Volume\left(V\right)=Length\left(L\right)\times Width(W{)^\wedge2}\times0.5$$

Tumour weight (g) was measured upon mice culling.

For the SCID mouse model, KPC WT, PAK4KO and PAK1&4 KO cancer cells (5 × 10^5^ cells/mouse) were injected subcutaneously into the flank of 6-week-old male SCID mice. Mice were monitored for the time indicated in the Result section. Tumour growth and weight were obtained as described above.

### Patient information collection and tissue microarray generation

The collection of patient’s clinical information and generation of human PDA tissue microarray (TMA) were approved by the Austin Health Human Research Ethics Committee (HREC/73948/Austin- 2021).

Patients who had surgical resection of PDA between 2008 and 2019 under Austin Health were identified. Patients who had premature mortality from either delayed surgical complications, background comorbidities, incorrect disease staging or missing survival data due to transfer of care were excluded. Once recruited, participants’ baseline demographics, disease staging and grading, treatment and survival information were retrospectively collected. The formalin-fixed PDA tumour blocks were assessed by a qualified anatomical pathologist, and three 1 mm diameter cores were taken from each tumour sample to assemble TMA blocks.

### CRISPR-CAS gene knock-out

To generate PAK4KO PDA cell lines, an inducible lentiviral CRISPR/Cas9 system was used as described previously [19]. Single-guide RNA (sgRNA) oligos targeting mouse PAK4 (guide 1: CCCGCGATAAGCGCCCACT; guide 2: CGAACGATGGTCTGGGGTC) were cloned into the BsmBI site of the pFgH1tUTG GFP lentiviral vector. KPC WT and KPC PAK1KO cells were infected with lentiviral constructs encoding Cas9 and mCherry, and a doxycycline-inducible sgRNA targeting PAK4 and GFP. mCherry and GFP double-positive cells were sorted using BD FACS Aria III (BD Biosciences, New Jersey, USA). Cell clones with PAK4KO were confirmed by immunoblot.

### Flow cytometry analysis

Mice tumour specimens were minced and digested in a digestion buffer containing 1.25 mg/ml collagenase IV (Worthington Biomedical, Lakewood, USA). Digested tumours were filtered and resuspended in FACS buffer to obtain single-cell suspension (Table S1). 1X10^6^ cells per sample were blocked with 1 μl of mouse FcR blocking reagent (Miltenyi, Biotec, Bergisch Gladbach, Germany). Cell viability was assessed using 1:500 dilution of Zombie UV™ Fixable Viability Dye (BioLegend, San Diego, USA). Fluorophore-labelled antibodies against CD45, B220, CD3, CD4, CD8, and PD1 (Table S3) were added and incubated on ice for 20 min.

For FoxP3 staining, cells were permeabilized and fixed with eBioscience™ FoxP3/Transcription Factor Staining Buffer Set (Invitrogen, Waltham, USA) according to the manufacturer’s instructions. Cells were incubated in FoxP3 fixation/permeabilization solution for 60 min in the dark at room temperature (RT). Fluorophore-labelled FoxP3 antibody was added to permeabilized cells and incubated for 30 min in the dark at RT (Table S3).

For staining of cytoplasmic markers, cells were permeabilized and fixed with eBioscience™ Intracellular Fixation and Permeabilization Buffer Set (Invitrogen, Waltham, USA) according to the manufacturer’s instruction. Cells were incubated in a fixation buffer for 60 min in the dark at RT. Fluorophore-labelled Granzyme B and Perforin antibodies were added to permeabilized cells and incubated for 30 min in the dark at RT (Table S3).

Cells were resuspended in FACS buffer and analyzed by Cytek® Aurora flow cytometer (Cytek Biosciences, California, USA). FCS Express version 7.12.0007 (De Novo Software, Pasadena, USA) was used for manual gating and statistical analysis.

### Global and phospho-proteomic studies

The proteomic studies were conducted consequently by these steps as *Sample Preparation,* *Liquid Chromatograph Data Independent Acquisition Mass Spectrometry*, *Data Search* and *Bioinformatic Analysis,* which were detailed in the Supplementary Methods.

### Analysis of TMA samples

The TMA blocks of 5 μM sections were stained using immunohistochemistry. Samples were boiled in Tris–EDTA buffer (Table S1) at 99˚C for 30 min and then blocked with Dako REAL™ peroxidase blocking solution (Agilent Technologies, Glostrup, Denmark) at RT for 15 min followed by 5% goat serum at RT for an hour for endogenous peroxidase quenching and protein blocking respectively. Primary antibodies against PAK1, PAK4, CD4 and CD8 (Table S2) were added to samples and incubated at RT for an hour before one hour incubation with Dako EnVision + System HRP Labelled Anti-Rabbit Polymer (Agilent Technologies, Glostrup, Denmark) at RT. DAB staining was achieved with EnVision FLEX DAB + substrate Chromogen System (Agilent Technologies, Glostrup, Denmark) and samples were counterstained with haematoxylin (Sigma-Aldrich, St Louis, USA). Whole slide images were captured with Aperio AT2 digital pathology slide scanner (Leica Biosystems, Wetzlar, Germany), and analysed by QuPath version 0.4.3 [20].

PAK1 and PAK4 expression were determined using mean DAB intensity of the TMA core, while CD4 + and CD8 + T cell levels were assessed by the percentage of positive stained cells against the total number of cells in the TMA core. Mean of all three replicates was calculated for each patient for PAK1, PAK4, CD4 and CD8 levels. Univariate and multivariate linear regression was used to assess correlation between individual variables and percentage of CD4 + or CD8 + cells. Correlation between individual variables and overall survival of patients was visualised with Kaplan Meier curve and tested by univariate and multivariate Cox proportional hazards regression. Interaction term was included for multivariate regression models to assess interaction between PAK1 and PAK4. Regression analysis was conducted using R version 4.3.0 (R Foundation for Statistical Computing, Vienna, Austria).

### Statistical analysis

All in vitro experiments were repeated in three replicates. In vivo experiments included at least three mice per group. For continuous variables, mean ± standard deviation (SD) was reported for parametric data while median ± inter-quantile range (IQR) were reported for non-parametric data. Hypothesis testing was conducted by either two-sided t test or one way ANOVA for parametric data, Mann–whitney’s U test for non-parametric data and Chi-square test for categorical results. Linear regression model was fitted for cell and tumour growth curve, and correlation coefficients (i.e. slopes) of linear fit was compared between groups. Statistical analysis was conducted with GraphPad Prism version 10.0.2 (GraphPad Software, Boston, USA), Stata BE version 17.0 (Texas, USA) and R version 4.3.0. *p*-value < 0.05 was considered statistically significant.

## Results

### Inhibition of PAK1 and PAK4 synergistically suppressed PDA growth

KPC WT, PAK1KO, PAK4KO and PAK1&4 KO cell lines were validated by immunoblot (Fig. S1a). PAK1KO reduced KPC cell growth by 72 h, but PAK4KO increased cell growth by 72 h (Fig. S1b). Global proteomic analysis of PAK WT and KO cells showed significantly differential protein expression profiles of PAK KO cell lines from WT cell line (Fig S2a-c). Functional enrichment for KEGG and reactome pathways indicated changes in programmed cell death (e.g., apoptosis) and cell cycle regulation (Fig. S2d, e), which was validated by FACS analysis. PAK1KO and PAK1&4 KO increased the apoptosis and cell death while PAK4KO reduced cancer cell death and had no effect on the apoptosis (Fig S1c, d).

**Fig. 1:**
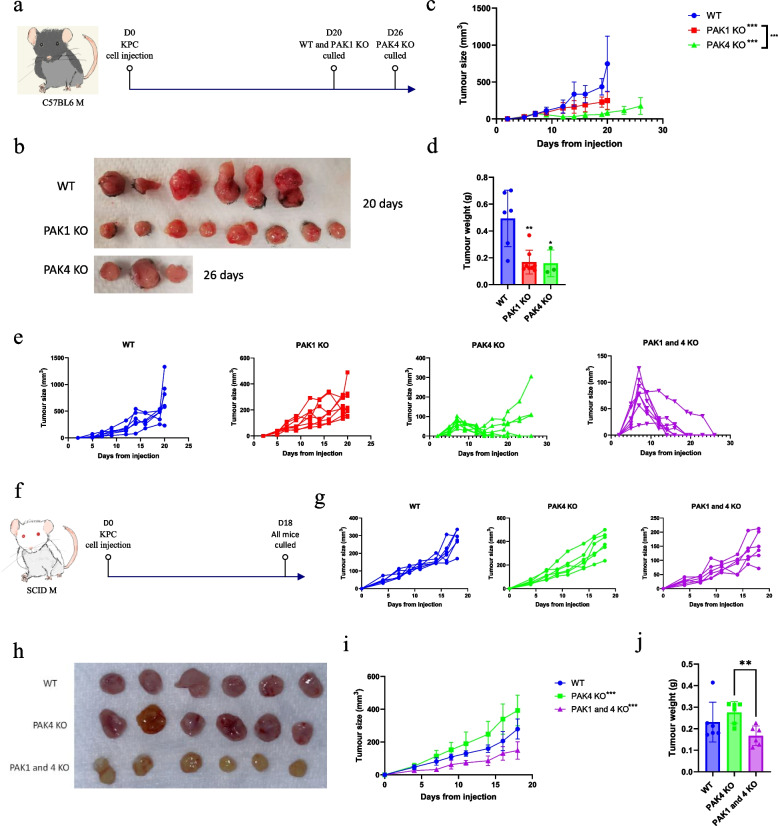
Knockout of PAK1 and PAK4 synergistically inhibited pancreatic cancer tumour growth. **a** C57BL6 mice were injected at right flank with KPC WT (*n* = 6), PAK1 KO (*n* = 8), PAK4 KO (*n* = 8) and PAK1 and 4 KO (*n* = 8) cell lines. Mice injected with WT and PAK1 KO cells were culled at day 20, while mice bearing PAK4 KO tumours were culled at day 26. Tumour photos **b**, size **c** and tumour weight **d** of KPC WT, PAK1 KO and PAK4 KO tumours were presented (PAK1 and 4 KO tumour regressed completely after two weeks). **E** Individual growth curve of KPC WT, PAK1 KO, PAK4 KO and PAK1 and 4 KO tumours in C57BL6 mice. **F **SCID mice were injected at left sided abdomen with KPC WT (*n* = 6), PAK4 KO (*n* = 6), PAK1 and 4 KO (*n* = 6) cell lines. Mice were culled at day 18. **G** Individual growth curve of KPC WT, PAK4 KO and PAK1 and 4 KO tumours in SCID mice. **H-j** Tumour photos, growth curve and tumour weight of KPC WT, PAK1 KO and PAK1 and 4 KO tumours in SCID mice. Statistical significance: *p < 0.05, **p < 0.01, ***p < 0.001. All comparisons were made against WT unless otherwise indicated

In a syngeneic mouse model (Fig. 1a), PAK4KO suppressed the in vivo tumour growth (Fig. 1a-d) significantly. Only 30% of the mice injected with PAK4KO cells developed tumours, which also grew significantly slower than the KPC WT- and PAK1KO-inoculated tumours, even with an extra two-weeks’ growth (Fig. 1c-e). No tumour developed in mice injected with PAK1&4 KO cells. The growth curve (Fig. 1e) showed a peak around one week after cell injection in PAK4KO and PAK1&4 KO injected mice followed by tumour regression. This suggested that PAK4KO inhibits PDA tumour growth through modulating the adaptive immune response, which is confirmed in a SCID mouse model (Fig. 1f-j). In SCID mice, PAK4KO did not inhibit the tumour growth at all while PAK double KO decreased the tumour growth significantly compared to PAK4KO but not to WT (Fig. 1g-j). These results indicated that PAK4KO inhibited PDA by stimulation of anti-tumour immunity and that inhibition of PAK1 and 4 synergistically suppressed PDA progression.

### Inhibition of PAK1 and PAK4 synergistically increased intra-tumoral CD8 + T cell infiltration

**Fig. 2:**
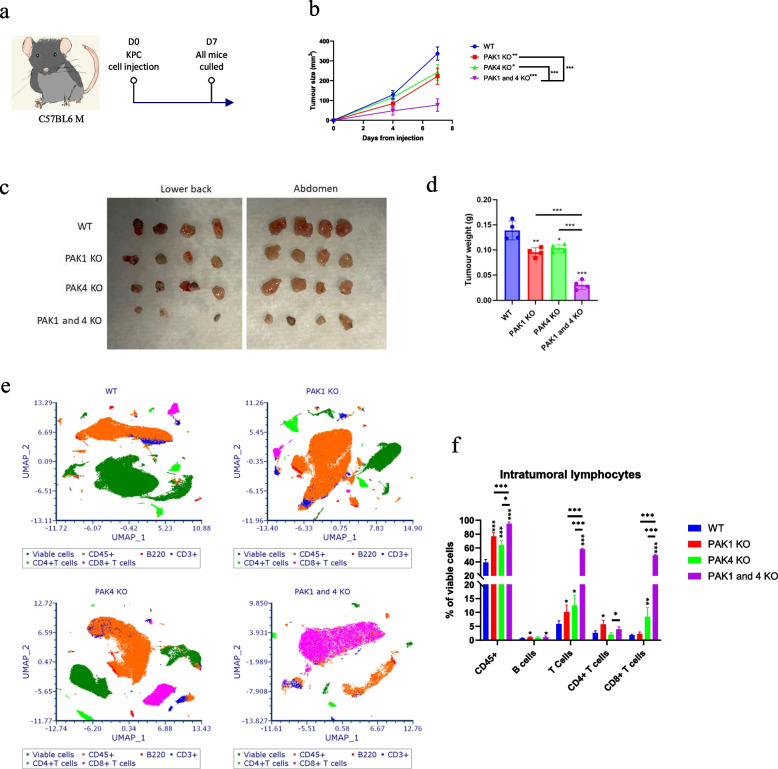
Knockout of PAK1 and PAK4 KO synergistically stimulated CD8 + T cell infiltration in pancreatic cancer. C57BL6 mice (4 in each group) were subcutaneously injected with KPC WT, PAK1 KO, PAK4 KO and PAK1 and 4 KO cell lines **a** at lower back and abdomen **c**. Mice were culled at day 7. Tumour size **b** and weight **d** were combined for lower back and abdomen tumours from each mouse. Tumour tissues collected on mice culling were subjected to FACS analysis for tumour infiltrating lymphocytes. **e** The infiltration of B cell (B220 +), CD4 + and CD8 + T cells in WT, PAK1 KO, PAK4 KO and PAK1 and 4 KO tumours were demonstrated by UMAP. **f** The percentage of cell infiltrations were compared. Statistical significance: *p < 0.05, **p < 0.01, ***p < 0.001. All comparisons were made against WT unless otherwise indicated

Given the findings that PAK4 KO tumour regressed one week after cell injection, we further examined PAK WT vs KO tumour growth within the first week (Fig. 2a, c). Tumour growth was inhibited by PAK1KO, PAK4KO and PAK1&4 KO (Fig. 2b, d). By one week, PAK double KO further suppressed the tumour growth compared to single PAK KO, indicating a synergistic effect of PAK1 and PAK4. One week tumour specimens were then digested into single cell suspension for FACS analysis of intra-tumoral lymphocyte infiltration (gating strategy shown in Fig. S4). By one week, PAK1KO increased B cell and CD4 + T cell infiltration while PAK4KO increased the infiltration of T cells, particularly CD8 + T cells (Fig. 2e, f). PAK1&4 KO further increased total T cell and CD8 + T cell infiltration, compared to single PAK4KO, suggesting a synergistic effect of PAK1KO and PAK4KO on intra-tumoral CD8 + T cell infiltration.

PAK4KO also increased the infiltration of active CD8 + T cells at one week, shown by increased levels of granzyme B + , and granzyme B and perforin double positive cytotoxic CD8 + T cells (Fig.S5a, b). However, Granzyme B + CD8 + T cells were reduced by PAK1KO, contributing to no change in granzyme B + CD8 + T cells and less increase in granzyme B and perforin double positive cytotoxic CD8 + T cells in double KO tumour (Fig. S5a, b). The level of granzyme B + CD4 + T cells was increased in both single PAK KO tumours, with a further significant increase in PAK1&4 double KO (Fig. S5c, d). The granzyme B and perforin double positive CD4 + T cells were increased only in PAK1&4 double KO tumour. These data suggested that PAK1 and PAK4 inhibition have synergistic effects on cytotoxic CD8 + and CD4 + T cell infiltration [21]. PAK4KO also decreased the infiltration of regulatory CD4 + T cells (Treg) (Fig. S5e, f). PAK1KO decreased PD-1 + CD8 + T cells while PAK4KO increased PD1 + CD8 + T cells, and PD1 + T cells were known to have higher anti-tumour activity (Fig. S5g, h) [22, 23].

### Pancreatic cancer developed immune evasion after PAK4 inhibition

**Fig. 3:**
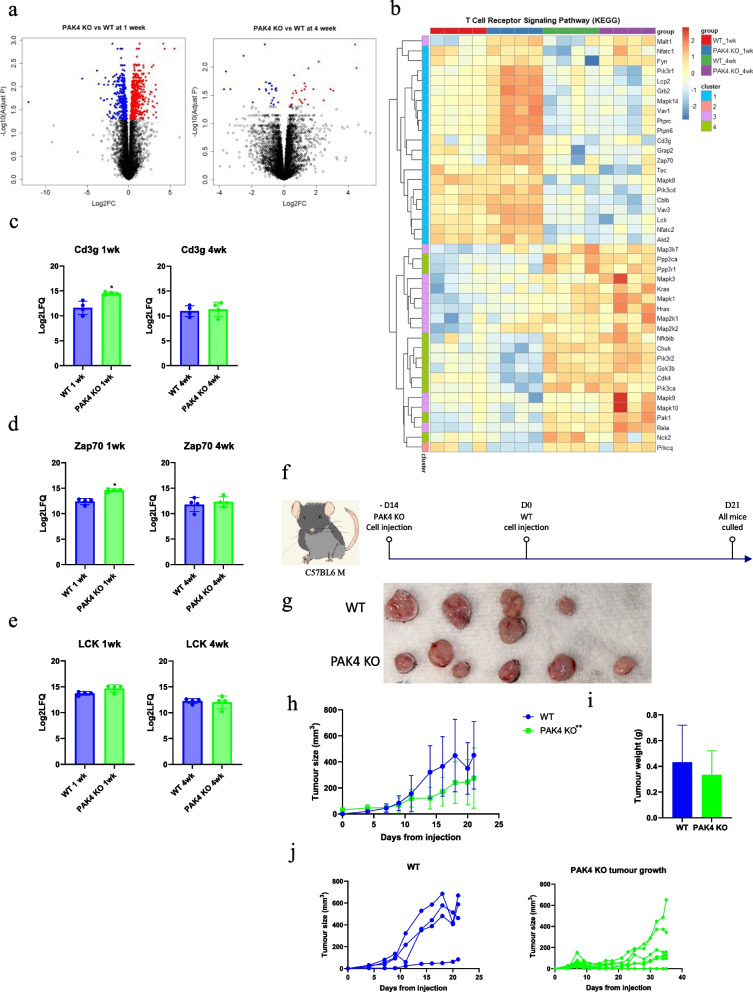
Immune evasion developed in PAK4 KO tumour. A global proteomic analysis of the tumour tissues of WT and PAK4KO showed that more differentially expressed proteins between PAK4 KO and WT tumours at 1 week than 4 weeks **a**. **b** Heatmap demonstrated individual protein expression identified in T cell receptor signaling KEGG pathway. The expression of CD3g **c**, Zap 70 **d** were significantly increased in PAK4KO tumour at 1 week but not at 4 weeks. There was a trend of increment of LCK **e** in PAK4 KO tumour at 1 week rather than at 4 weeks. **f** C57BL6 mice were injected at right flank with KPC WT (*n* = 4), PAK4KO (*n* = 16) cell lines. PAK4 KO cell line was injected 14 days before WT cell line injection (D0) and only five mice developed PAK4KO tumour. Mice were culled at day 21. **g-i** PAK4 KO tumours that escaped immune surveillance showed similar growth as WT tumours demonstrated by tumour photos **g**, growth curve **h** and tumour weight **i**. **j** Individual growth curve of KPC WT and PAK4 KO tumours. Statistical significance: *p < 0.05, **p < 0.01, ***p < 0.001. All comparisons were made against WT unless otherwise indicated

After initial regression, approximately 30% of mice developed a PAK4KO tumour (Fig. 1e), indicating immune evasion. We conducted a proteomic study to compare PAK4KO versus WT tumours at one week and four weeks after cancer cell injection to investigate the differences in immune cell infiltration. Protein expressions of PAK4KO and WT tumours were much more different at one week than four weeks (Fig. 3a). Four protein clusters were classified based on expression patterns and were visualized as heatmap and UMAP (Fig. S6a, b). All four clusters of proteins had different protein expression levels between PAK4 KO and WT tumours at one week, and these differences were reduced in clusters 1, 2 and 4 at four weeks (Fig. S6a). KEGG and reactome pathways were functionally enriched based on the significant proteins and identified immune-related pathways (Fig. S6c, d).

Given that the data from FACS analysis showed a significant role of PAK4KO in CD8 + T cell mediated immune response (Fig. 2), T cell receptor signaling pathway was examined in greater detail by heatmap. Multiple protein targets that are known to play an important role in downstream signaling of T cell receptor (TCR) were identified (Fig. 3b), such as the VAV family proteins (VAV1, VAV3) and proteins that are directly involved in MHC- TCR signaling complex (CD3g, ZAP70, LCK) [24, 25]. The expression profiles of CD3g, ZAP70 and LCK were compared between WT and PAK4KO at one week and four weeks. CD3g and ZAP70 levels were significantly higher in PAK4KO tumour at one week but dropped to similar levels as WT tumour by four weeks (Fig. 3c, d). There was no difference in LCK levels between WT and PAK4 KO tumours at both time points (Fig. 3e).

These findings from global proteomic analysis were further supported by phosphor-proteomic results. Differentially expressed phospho-sites between WT and PAK4 KO tumours were demonstrated at one and four weeks (Fig. S6e). From the significant phospho-sites identified across all four groups, 38 kinases and their activity were predicted (Fig. S6f). Among these predicted kinases, IKKε (IKBKE) and PKCθ (PRKCQ) were predicted to have significantly higher activity in PAK4 KO tumour at one week in comparison to WT tumour (Fig. S6g) indicating a higher activity of T cell receptor pathway in PAK4KO as both IKKε and PKCθ are activated upon activation of T cell receptor [26–28]. However, by four weeks, the kinase activities of IKKε and PKCθ were no longer different between PAK4 KO and WT tumours (Fig. S6h) suggesting an immune evasion of PAK4KO tumour after the initial response. This is confirmed by the finding that PAK4KO tumour grew at a similar pace to WT after escaping the initial regression (Fig. 3f-j). The PAK WT cancer cells were injected two weeks after PAK4KO cells as PAK4KO tumour grew out of the initial regression after two weeks. When comparing the PAK4KO tumour with WT tumour, there was no difference in tumour weight (Fig. 3i) though PAK4KO tumour still demonstrated a slightly slower growth rate in comparison to WT in tumour size (Fig. 3h). These results suggested that PAK4KO tumour developed an immune evasion after an initial phase due to a reduced immune response induced by PAK4KO from one week to four weeks.

### PAK1 inhibition induced CD8 + T cell infiltration at a later phase

**Fig. 4:**
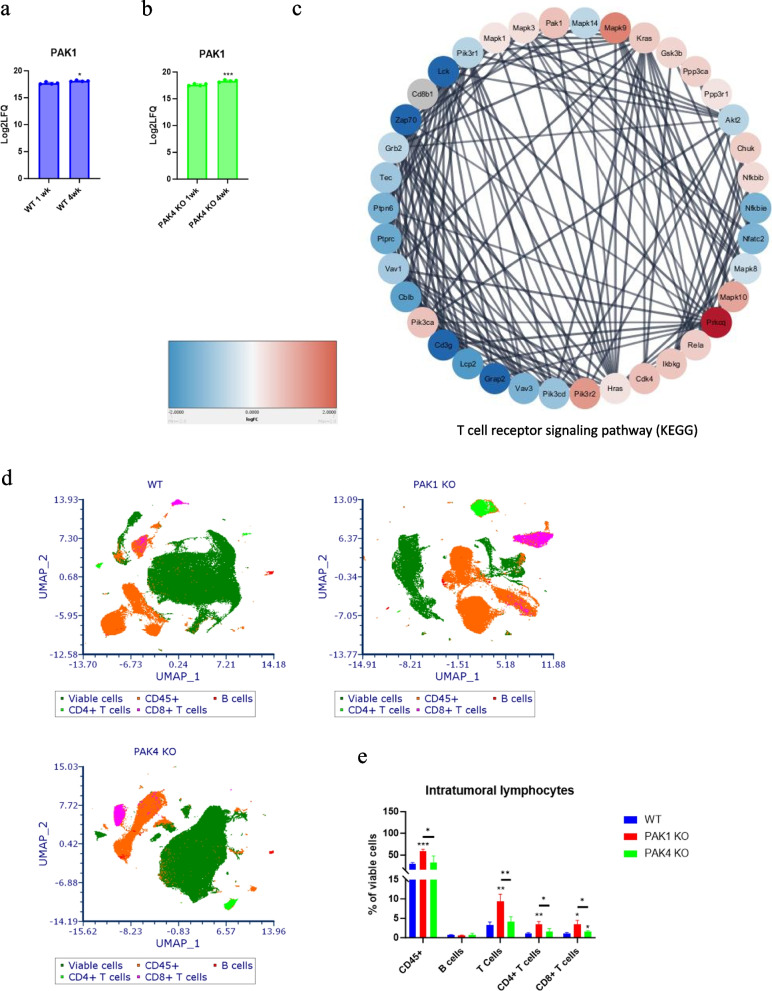
PAK1 KO induced delayed CD8 + T cell infiltration. **a-b** PAK1 expression increased from 1 to 4 weeks in both WT and PAK4 KO tumour from global proteomic analysis, but to a higher degree in PAK4 KO tumours. **c** Protein–protein interaction network analysis of differentially expressed proteins of PAK4 KO tumours between 4 weeks and 1 week showed a role of PAK1 in T cell receptor signaling. Tumour specimens collected from the experiment described in Fig. 1a were subjected to FACS. The infiltration of B cell (B220 +) and CD4 + T cell were significantly increased in PAK1 KO tumour rather than PAK4 KO tumour **d**, **e**. Although the infiltration of CD8 + T cell was still significantly increased in PAK4KO tumour **d**, **e**, its level has reduced in comparison to one week results (Fig. 2e,f). Statistical significance: *p < 0.05, **p < 0.01, ***p < 0.001. All comparisons were made against WT unless otherwise indicated

Global proteomic analysis of PAK WT and KO tumours also identified an up-regulation of PAK1 expression from 1 to 4 weeks in both WT and PAK4KO tumours, but slightly greater in PAK4KO tumour (Fig. 4a, b). Functional enrichment of PAK4KO identified differential expression of proteins involved in T-cell receptor signalling pathways between one week and four weeks. PPI network was constructed and annotated by log2(FC) of individual proteins. VAV family proteins, CD3g, ZAP70 and LCK were down-regulated at four weeks in comparison to one week (Fig. 4c), suggesting a downregulation of T-cell response in PAK4KO tumour over time. Furthermore, the upregulation of PAK1 over four weeks was found to be involved in T-cell receptor signaling pathways (Fig. 4c), indicating that the increase of PAK1 level in PAK4KO tumour over time may contribute to their immune resistance.

PAK4KO tumour still had slightly higher CD8 + T cell infiltration than WT tumour by four weeks, but the difference was a lot less compared to the increase at one week (Figs. 2 e, f, and 4d, e). Furthermore, the percentage of total T cells (CD45 + CD3 +) in PAK4KO tumour was no longer different to WT tumour after four weeks. On the other hand, PAK1KO tumour demonstrated a significant increase of CD8 + T cell infiltration by three weeks (Fig. 4d, e) compared to at one week (Fig. 2e, f), suggesting a delayed CD8 + T cell response in PAK1 KO tumour. In addition, PAK4KO reduced the percentage of Treg and increased the level of PD1 + CD8 + T cells at one week (Fig. S5e-h), which were reversed by four weeks (Fig. S7a-d), suggesting a more immunosuppressive environment. On the other hand, although PAK1KO had a high level of Treg (Fig. S7a, b), it also had an increase in PD1 + CD8 + T cell level (Fig. S7c, d) from one week to three weeks, suggesting that CD8 + T cells had increased anti-tumour activity in PAK1KO tumour over time. Together these results indicated that PAK1KO caused a delayed infiltration of CD8 + T cells which may compensate for the reduced immune response to PAK4KO over time.

### Low PAK1 and PAK4 expressions improved T cell function in human pancreatic *cancer*

To study the roles of PAK1 and PAK4 in human PDA, 178 PDA cases were assessed from the TCGA database. High PAK1 and PAK4 gene expressions were associated with reduced survival in PDA patients (Fig. S8a, b). Functional enrichment of genes correlated with PAK1 (Fig. S8c) and PAK4 (Fig. S8d) revealed immune-related pathways for PAK4 but not PAK1. Tumour purity adjusted estimation of intra-tumoral B and T cell infiltration showed a negative correlation between PAK4 and CD8 + T cell infiltration, but not with B cells or CD4 + T cells (Fig. S8f). No correlation between PAK1 and lymphocyte infiltration was demonstrated (Fig. S8e).

**Fig. 5:**
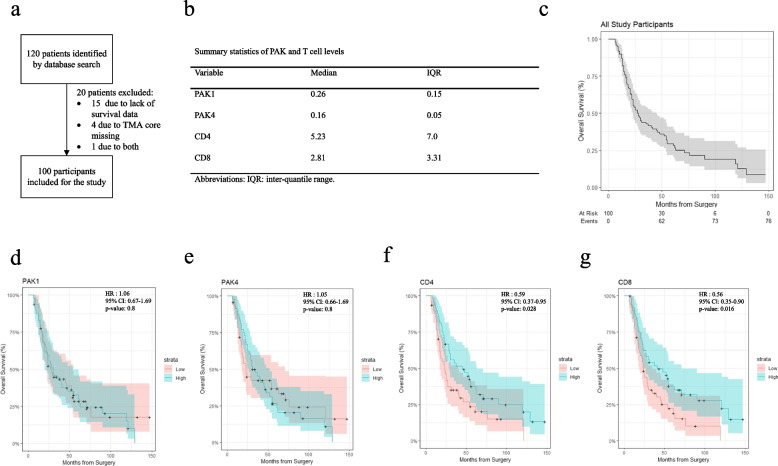
Correlation of PAKs and T cells to the survival of pancreatic cancer patients. The data were obtained from a tissue microarray (TMA) study from human pancreatic cancer patients (*n *= 100). **a** Flow diagram demonstrating identification, exclusion, and inclusion of study participants. **b** Table of median PAK1, PAK4, CD4 and CD8 levels as well as inter-quantile range (IQR). **c** Kaplan-meier curve demonstrating overall survival of all study participants, as well as number of participants at risk at each time point. **d-g** Kaplan-meier curves demonstrating correlation between PAK1, PAK4, CD4 and CD8 levels and overall survival respectively. Hazard ratio (HR), confidence interval (CI) and *p*-values were reported for all survival analysis, with two-sided p-value below 0.05 considered statistically significant

**Fig. 6:**
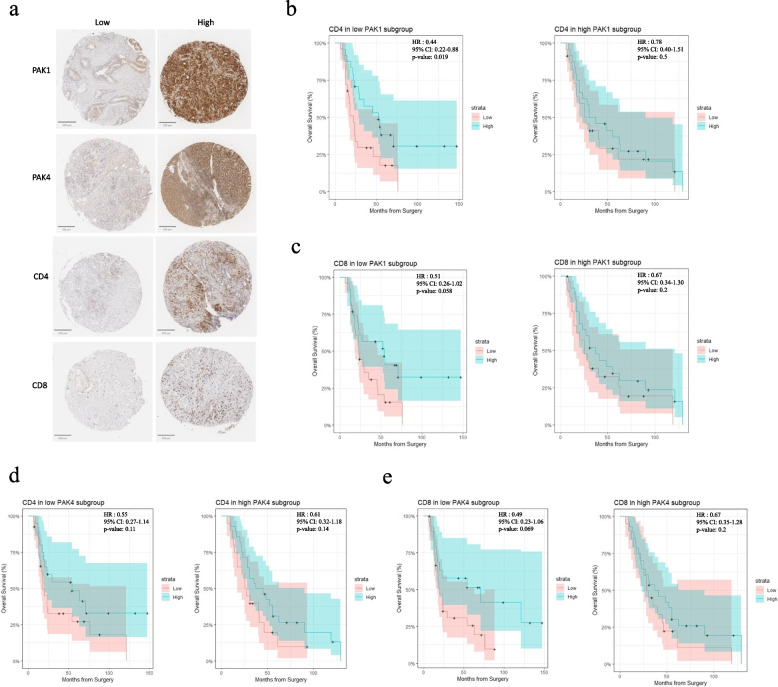
Impact of PAK levels on the effects of T cells on patients’ survival. The data were obtained from a tissue microarray (TMA) study from human pancreatic cancer patients (*n* = 100). **a** Immunohistochemistry (IHC) staining of low versus high PAK1, PAK4, CD4 and CD8 TMA cores. Kaplan-meier curve demonstrated the correlation between CD4 **b** or CD8 **c** levels to the overall survival in low versus high PAK1 expression subgroups. Similarly, Kaplan-meier curve demonstrated the correlation between CD4 **d** or CD8 **e** levels to the overall survival in low versus high PAK4 expression subgroups. Hazard ratio (HR), confidence interval (CI) and *p*-values were reported for all survival analysis, with two-sided *p*-value below 0.05 considered statistically significant

In a TMA analysis of PDA patients (Fig. 5a), patients were grouped into low or high expression groups based on median of PAK1 or PAK4 intensity as well as median percentage of CD4 + or CD8 + positive cells (Figs. 5b and 6a). Individual study variables were compared between low versus high expression groups for PAK1 and PAK4 (Table S4 and S5). To assess the relationship between PAK expression and the level of T cell infiltration, both univariate and multivariate linear regression models were applied (Table S6 and S7). By univariate analysis, PAK4 expression was positively correlated with CD4 + and CD8 + T cell infiltration and PAK1 expression was positively correlated with CD4 + T cell infiltration. However, these correlations disappeared in multivariate analysis (Table S6 and S7).

Overall survival of patients was demonstrated by Kaplan Meier curve (Fig. 5c), with a number of patients at risk and events reported. While high levels of CD4 + and CD8 + T cell infiltration significantly improved overall survival (Fig. 5f, g), PAK1 and PAK4 expression levels did not significantly affect patient survival by univariate analysis (Fig. 5d, e). However, high PAK4 levels increased risk of death with multivariate analysis (Table 1). Furthermore, PAK1 and PAK4 demonstrated negative interaction (HR below 1), indicating that a reduction in the level of either PAK1 or PAK4 would lead to worse survival outcome for the other (Table 1).

**Table 1:** Univariate and multivariate Cox regression for survival analysis

	Univariate		Multivariate	
Variable	Hazard ratio (HR)	*p*-value	Hazard ratio (HR)	*p*-value
Age	1	0.7	1.05	0.005**
Sex				
- Male	-	-	-	-
- Female	0.89	0.6	0.80	0.5
PAK1				
- Low	-	-	-	-
- High	1.06	0.8	1.39	0.5
PAK4				
- Low	-	-	-	-
- High	1.05	0.8	2.58	0.04*
PAK1: PAK4 interaction	-	-	0.2	0.021*
CD4				
- Low	-	-	-	-
- High	0.59	0.028*	0.35	0.003**
CD8				
- Low	-	-	-	-
- High	0.56	0.016*	1.15	0.7
Cancer Site				
- Head/neck	-	-	-	-
- Body/tail	0.46	0.092	2.31	0.2
- Multifocal	2.13	0.5	2.67	0.4
Resection Margin				
- R0	-	-	-	-
- R0	2.02	0.003**	2.95	0.003**
T stage				
- 1	-	-	-	-
- 2	2.9	0.009**	6.22	0.002**
- 3	4	0.003**	7.57	0.002**
- 4	3.79	0.2	42.6	0.006**
N stage				
- 0	-	-	-	
- 1	1.24	0.5	1.47	0.3
- 2	1.67	0.078	2.15	0.052
M stage				
- 0	-	-	-	-
- 1	24.2	0.004**	98.8	< 0.001***
Grade				
- 1	-	-	-	-
- 2	2.08	0.3	0.59	0.7
- 3	3.56	0.081	1.63	0.7
Lymphovascular Invasion				
- Yes	-	-	-	-
- No	0.72	0.2	0.88	0.7
Perineural Invasion				
- Yes	-	-	-	-
- No	0.68	0.11	2.11	0.035*
Adjuvant chemotherapy				
- Yes	-	-	-	-
- No	1.77	0.045*	2.01	0.11

*Abbreviations*: *CI* Confidence interval

Statistical significance: *p* < 0.05^*^, *p* < 0.01^**^, *p* < 0.001^***^

Finally, subgroup analysis of overall survival was conducted for levels of CD4 + and CD8 + T cells based on PAK1 or PAK4 expression. While high levels of CD4 + or CD8 + T cells failed to improve patient survival in the high PAK1 expression group (Fig. 6b, c), high level of CD4 + T cells significantly improved overall survival in the low PAK1 subgroup (Fig. 6b). There was also a trend suggesting high level CD8 + T cells was associated with better survival (p = 0.058) in low PAK1 patients (Fig. 6c). On the other hand, PAK4 expression level did not affect the correlation between CD4 + T cells and patient survival (Fig. 6d). However, despite not being statistically significant, the low PAK4 expression level resulted in a trend (*p* = 0.069) of improved overall survival by CD8 + T cells (Fig. 6e).

## Discussion

The individual roles of PAK1 and PAK4 in PDA tumorigenesis have been recognized in the literature [29]. However, the development of selective PAK1 or PAK4 inhibitors for the treatment of solid tumours has not been successful [29, 30]. The fact that both PAK1 and PAK4 have been implicated in intra-tumoral T-cell response, suggested that PAK1 and PAK4 inhibition may function synergistically in suppressing the growth of PDA [10, 16, 31]. In this study, we demonstrated a synergistic effect of PAK1 and PAK4 inhibition in suppressing PDA growth in mice. PAK4KO stimulated the infiltration and activation of CD8 + T cells in the tumour to a greater degree at an initial phase while PAK1KO caused an increased infiltration of active CD8 + T cells at a late phase. Together PAK1 and PAK4 double KO stimulated a sustained increase of infiltration of active CD8 + T cells, leading to a complete tumour regression. The results from a TMA of human PDA also confirmed the importance of PAK1 and PAK4 in intra-tumoral CD4 + and CD8 + T cell function, and the impact on overall survival of PDA patients.

The fact that PAK4KO suppressed tumour growth in a syngeneic mouse model but not in SCID mice, indicates that PAK4KO regresses tumour growth through stimulating the anti-tumour immunity. An increased infiltration of cytotoxic CD8 + T cells was demonstrated in the PAK4KO PDA tumour, which is consistent with the results from melanoma and prostate cancer [15, 18]. However, an immune evasion developed in the PAK4KO tumour (Fig. 3f-j) due to reduced infiltration and activation of CD8 + T cells at a later phase (four weeks, Fig. 4d, e). While PAK1KO did not enhance CD8 + T cell infiltration significantly at the initial phase (one week, Fig. 2e,f), it caused a delayed increase in the infiltration of CD8 + T cells (Fig. 4d,e), which is consistent with our previous findings in a syngeneic mice model [10]. This delayed effect of PAK1KO is likely to compensate for the reduced anti-tumour immune response by PAK4KO over time, leading to a sustained immune response to kill cancer cells. Therefore, the tumour was completely regressed by PAK1 and PAK4 double knockout (Fig. 1e).

While high PAK1 expression was previously shown to be negatively correlated with PDA patient survival, this was not found in our results from the human PDA TMA dataset [10]. After correcting for tumour stage, grade, and resection status, high PAK4 expression was noted to be associated with worse outcomes which is the opposite of a previous report [32]. We also found a positive correlation between CD4 + or CD8 + T cell infiltration and PDA patient survival. Furthermore, low PAK1 or PAK4 levels enhanced the pro-survival effects of CD4 + and CD8 + T cells. This again highlights the important roles of PAK1 and PAK4 in T cell response in human PDA.

Our findings warrant further studies to validate the combined effects of PAK1 and PAK4 on the tumour immune response of PDA in orthotopic mouse models where the biology of pancreatic cancer gets assessed anatomically, and in KPC mice with conditional knockout of PAK1 and/or PAK4 although it is challenging to generate these mice. More importantly, our findings lay down a solid base for further testing the combined effects of PAK1 and PAK4 on the tumour immune response of PDA using selective PAK inhibitors, which will provide a clear guide for the clinical translation of this basic research work.

The high homology among the members of the PAK family makes it difficult to develop highly selective PAK inhibitors. The cross-reactive and off-target effects of the existing PAK inhibitors contribute to the fact that there have been no successful clinical trials in testing PAK inhibitors. Advanced knowledge and techniques are required to develop highly selective and fewer side-effects of PAK inhibitors for biological tests.

## Conclusion

Our results identified a synergistic effect of PAK1 and PAK4 inhibition on PDA growth and T-cell immune response. It also indicated a rapid development of immune evasion with selective PAK4 inhibition in PDA, which may explain the failure of selective PAK4 inhibitors in clinical trials. However, pan-PAK inhibitors have a high level of toxicity as PAK2 inhibition can lead to endothelial cell dysfunction and vascular malformation [33]. Recent development of a selective PAK1 degrader by proteolysis-targeting chimera (PROTAC) technique has increased the hope of developing more selective PAK inhibitors [34]. Whether the combination of selective PAK1 and PAK4 degraders can offer greater clinical efficacy while minimizing side effects remains a question to be addressed and requires further research.

### Supplementary Information

Supplementary Material 1.

Supplementary Material 2.

Supplementary Material 3.

## Data Availability

No datasets were generated or analysed during the current study.
