# Supplementary material for: Inhibition of P21-activated kinases 1 and 4 synergistically suppresses the growth of pancreatic cancer by stimulating anti-tumour immunity
Source: Cell Commun Signal. 2024 May 27;22:287. doi: 10.1186/s12964-024-01670-2 (PMC11129409; doi:10.1186/s12964-024-01670-2)
Supplement: Supplementary file 1 — Supplementary Material 1. [file 12964_2024_1670_MOESM1_ESM.docx]

**Supplementary Materials and Methods**

## Global and phospho-proteomic studies

### *Sample Preparation*

Tumour tissue was lysed in RIPA buffer containing PhosSTOP phosphatase inhibitor (Roche, Mannheim, Germany) and Complete Mini protease inhibitor (Roche, Mannheim, Germany), followed by sonication to improve digestion. KPC WT, PAK1KO, PAK4KO and PAK1&4 KO cells were seeded into 10 cm culture dishes and grown until 80% confluency. Cells were lysed in RIPA buffer containing PhosSTOP phosphatase inhibitor and Complete Mini protease inhibitor. Supernatant was collected and precipitated with ice-cold acetone overnight at -20^o^C before resuspension in 8 M urea (in 50mM triethylammonium bicarbonate + 10mM TCEP) followed by incubation at 37^o^C for 30mins. Protein concentration was quantified using BCA protein assay and samples (containing equivalent amounts of total protein) were alkylated with 55 mM iodoacetamide for 45 mins at RT, followed by overnight digestion at 37^o^C with sequencing-grade modified trypsin (Thermofisher) at a ratio of 1:50 (enzyme:protein). Digestion was stopped by addition of formic acid at a final concentration of 1% (v/v). The peptide mix was purified through solid phase extraction (SPE) with Oasis HLB cartridges (Waters) according to manufacturer’s instructions. Eluted peptides were split into two tubes, Tube 1 (global mass spec) and Tube 2 (phospho-enrichment) and freeze-dried. Tube 1 was resuspended in 2% acetonitrile and 0.05% trifluoracetic acid for mass spectroscopy. Phosphorylated peptides from Tube 2 were purified using a titanium dioxide (TiO_2_) enrichment method as previously reported[1]. Peptides were mixed at a ratio of 1:6 (peptides: TiO_2_ beads) with TiO_2_ beads (GL sciences) in loading buffer (2M Lactic acid in 5% trifluoroacetic acid (TFA), 50% acetonitrile) and incubated for 1 hour at RT. The beads were washed twice with loading buffer and then three times with washing buffer (50% acetonitrile, 5% TFA). The phosphorylated peptides were eluted with 1% (v/v) ammonia solution (pH 11.3) and 30% (v/v) acetonitrile, and then acidified with 1μL formic acid per 10μL eluent. Eluted phosphorylated peptides were freeze-dried, resuspended with 2% acetonitrile and 0.05% TFA for mass spectroscopy.

### *Liquid Chromatograph Data Independent Acquisition Mass Spectrometry*

Liquid chromatograph triple quadrupole mass spectrometry (LC-MS/MS) was carried out using a Orbitrap Eclipse mass spectrometer (Thermo Scientific) equipped with a nanoflow reversed-phase-HPLC (Ultimate 3000 RSLC, Dionex) fitted with an Acclaim Pepmap nano-trap column (Dionex—C18, 100 Å, 75 µm× 2 cm) and an Acclaim Pepmap RSLC analytical column (Dionex—C18, 100 Å, 75 µm× 50 cm). The tryptic peptides were injected to the enrichment column at an isocratic flow of 5 µL/min of 2% v/v CH_3_CN containing 0.1% v/v formic acid for 5 min before the enrichment column was switched in-line with the analytical column. The eluents were 5% DMSO in 0.1% v/v formic acid (solvent A) and 5% DMSO in 100% v/v CH_3_CN and 0.1% v/v formic acid (solvent B). The flow gradient was (i) 0-6min at 3% B, ii) 6-7min, 3-4% (ii) 7-82 min, 4-25% B (iii) 82-86min 25-40% B (iv) 86-87min, 40-80% B (v) 87-90min, 80-80% B (vi) 90-91min, 80-3% and equilibrated at 3% B for 10 minutes before the next sample injection.

For data independent acquisition (DIA) experiments full MS resolutions were set to 120,000 at m/z 200 and scanning from 350-1400m/z in the profile mode. Full MS AGC target was 250% with an IT of 50 ms. AGC target value for fragment spectra was set at 2000%. 50 windows of 13.7 Da were used with an overlap of 1 Da. Resolution was set to 30,000 and maximum IT to 55 ms. Normalized collision energy was set at 30%. All data were acquired in centroid mode using positive polarity.

### *Database Search*

DIA data were analyzed using the direct DIA analysis workflow with default settings on the Spectronaut software (v. 17.5.230413.55965) and against the Uniport Mus Musculus database (updated Apr 2022, 17544 entries). Trypsin specificity was set to two missed cleavages. Carbamidomethyl (Cys) was defined as fixed modification and acetylation (protein N-term) and oxidation (Met) as variable modification. For phospho-proteomics, phosphorylation of serine, threonine and tyrosine were considered as additional variable modifications. Results are filtered at a Protein and PSM false discovery rate of 1%. Precursor filtering using the Qvalue, quantification carried out on the MS2 level, PTM localization set to on, and cross-run normalization strategy set to automatic.

### *Bioinformatic Analysis*

For global proteomic analysis, LFQ data was log2 transformed and filtered by 75% of valid entry per group. Missing values were imputed by scImpute and tail-based imputation, and data was scaled by median scaling[2]. Significant proteins were then identified by ANOVA with adjusted p-value less than 0.05 considered statistically significant. Number of protein clusters was estimated by elbow-method and K-means clustering was conducted. Dimension reduction was performed by Uniform Manifold Approximation and Projection (UMAP). Kyoto Encyclopedia of Genes and Genomes (KEGG) and Reactome pathways were then functionally enriched based on significant proteins by using STRING database version 12.0[3]. For each pathway, ratio of identified protein versus background protein in the pathway was calculated and -log10 of False Discovery Rate (FDR) was reported. Expression of significant proteins were then standardised across groups and visualised on heatmap. To compare protein expression between groups, linear model was fitted to log2(LFQ) data using limma package[4]. Moderated t-statistics, F-statistics and log odds of differential expression was computed by empirical Bayes moderation. Adjusted P-value as well as log2 of Fold Change (FC) between groups were computed, with adjusted P-value less than 0.05 considered significant. Protein-protein interaction (PPI) network was then constructed based on significant proteins with node colour annotated by log2(FC).

PhosR package was used for phospho-proteomic analysis and kinase prediction following the provided protocol[5, 6]. Like global proteomic analysis, phosphosite LFQ data was log2 transformed, filtered, imputed for missing values and scaled as described above[2]. For inter-group comparisons, linear model was fitted using limma package in the same way as global proteomic[4]. Adjusted P-value as well as log2(FC) were computed, and adjusted P-value less than 0.05 was considered significant. LFQ data was then converted to ratio (i.e. log2(Ratio)) against the means of the control group. ANOVA was applied and phosphosites with an adjusted p-value below 0.05 were considered significant. Kinases were then predicted based on significant phosphosites by computing motif-matching scores and profile-matching scores using the Mus Musculus dataset in the PhosphoSite Plus database[6, 7]. A heatmap was constructed to demonstrate the combined score (based on motif matching score and profile matching score) of top 3 phosphosites for each predicted kinase. Kinase activity as predicted for each sample based on Log2(Ratio) of phosphosites. Kinase activity was compared between groups by a linear model and empirical Bayes moderation as described above with log2(FC) reported an adjusted P-value less than 0.05 considered significant.

R version 4.3.0. and Cytoscape version 3.10.0 were used for statistical analysis, functional enrichment as well as construction of PPI network[8].

## Immunoblot

Cells were lysed with RIPA buffer containing PhosSTOP phosphatase inhibitor (Roche, Mannheim, Germany) and Complete Mini protease inhibitor (Roche, Mannheim, Germany). Protein concentration was quantified by DC protein assay (Bio-Rad, California, USA). Cell lysates were electrophoresed through 10% SDS gel before being transferred onto a nitrocellulose membrane which was blotted with primary antibodies against PAK1, PAK4, and GAPDH (Table S2) followed by goat anti-rabbit IgG(H+L)-HRP conjugate (Bio-Rad, California, USA). Images were detected with ECL Select^TM^ Detection Reagent (Cytiva, Amersham Place, UK) and captured by ChemiDoc^TM^ MP Imaging system (Bio-Rad, California, USA). Densitometry analysis of image was conducted by ImageJ2[9].

## MTT assay

WT, PAK1KO, PAK4KO and PAK1&4 KO KPC cells were seeded into 96-well plate at 5000 cells/well, and incubated for 4, 24, 48 and 72 hours. 10μl of 5mg/ml MTT (Thiazolyl Blue Tetrazolium Bromide, Sigma-Aldrich, St. Louis, USA) were added to culture media and incubated for 2 hours at 37˚C. After incubation, culture media was discarded, and cells were resuspended in 100μl of acidified isopropanol. Absorbance was measured at 570nm and 670nm wavelengths by FLUOstar OPTIMA microplate reader (BMG Labtech, Ortenberg, Germany).

## Cell death and cell cycle analysis

KPC WT, PAK1KO, PAK4KO and PAK1&4 KO cell lines were seeded at 1X10^5^ cells/well and cultured for 24 hours. Cells were then collected and resuspended in FACS buffer. APC Annexin V Apoptosis Detection Kit with 7-AAD (BioLegend, San Diego, USA) was used for detection of apoptotic cells, but 7-AAD was replaced by DAPI for viability staining. Cells were incubated with 1µl APC labelled Annexin V and 0.2 µg/mL of DAPI (BD Biosciences, Franklin Lakes, USA) in Annexin V binding buffer for 15 minutes at RT in dark before being subjectedto FACS analysis.

Cell cycle was assessed using Click-iT^TM^ Plus EdU Alexa Fluor^TM^ 647 Flow Cytometry Assay Kit (Invitrogen, Waltham, USA). KPC WT, PAK1KO, PAK4KO and PAK1&4 KO cell lines were seeded at 1X10^5^ /well and incubated for 24 hours in DMEM without serum. Culture medium was then changed to DMEM with 5% FBS and10μM EdU and incubated for two hours before being trypsinised and resuspended in 1X Phosphate Buffered Saline (PBS) with 1% Bovine Serum Albumin (BSA) (Sigma-Aldrich, St. Louis, USA). Cells were fixed using Click-iT fixative for 15 minutes at RT, and then permeabilized with 1X Click-iT permeabilization followed by washing for 15 minutes at RT in dark. Click-iT plus reaction cocktail was incubated with cells for 30 minutes at RT in dark. Cells were then stained with 1μg/ml of DAPI (BD Biosciences, Franklin Lakes, USA) for 5 minutes at RT.

Samples were analyzed by FACSymphony A3 flow cytometer (BD Biosciences, Franklin Lakes, USA). FCS express version 7.12.0007 (De Novo Software, Pasadena, USA) was used for manual gating and statistical analysis.

## TCGA database analysis

Gene expression profile and survival data of 178 human PDA cases were curated from The Cancer Genome Atlas (TCGA) database using R2: Genomic Analysis and Visualisation Platform (http://r2.amc.nl). A cut-off of 3368.7149 and 1770.4213 were used for gene expression of PAK1 and PAK4 to define high versus low expression cohorts respectively. Kaplan-Meier analysis of patient survival was performed based on the gene expression levels of PAK1 or PAK4, and statistically tested by log-rank test. A raw p-value below 0.05 was considered statistically significant. Genes with FDR below 0.05 and correlation coefficient (r) greater than 0 were considered significantly correlated with PAK1 or PAK4. Top 200 negatively correlated genes were then selected for functional enrichment analysis.

Functional enrichment analysis was conducted using Metascape platform[10]. Gene ontology (GO), KEGG, WikiPathways and Reactome pathways were enriched based on selected genes and top 20 pathways with highest -log10(P-value) were reported. Intra-tumoral infiltration of B cells, CD4+ and CD8+ T cells were estimated from RNA sequencing data of TCGA PDA cases using Tumour Immune Estimation Resource (TIMER) 2.0 platform[11, 12]. Correlation between PAK1 or PAK4 with intra-tumoral lymphocyte infiltration was assessed by linear model with adjustment against tumour purity. Spearman’s Rho correlation coefficient (Rho) and associated p-value were reported. A p-value below 0.05 was considered statistically significant.

# **Reference**

[1] H. He, C. Dumesny, C.S. Ang, L. Dong, Y. Ma, J. Zeng, M. Nikfarjam, A novel PAK4 inhibitor suppresses pancreatic cancer growth and enhances the inhibitory effect of gemcitabine, Transl Oncol, 16 (2021) 101329.

[2] W.V. Li, J.J. Li, An accurate and robust imputation method scImpute for single-cell RNA-seq data, Nat Commun, 9 (2018) 997.

[3] C. von Mering, M. Huynen, D. Jaeggi, S. Schmidt, P. Bork, B. Snel, STRING: a database of predicted functional associations between proteins, Nucleic Acids Res, 31 (2003) 258-261.

[4] M.E. Ritchie, B. Phipson, D. Wu, Y. Hu, C.W. Law, W. Shi, G.K. Smyth, limma powers differential expression analyses for RNA-sequencing and microarray studies, Nucleic Acids Res, 43 (2015) e47.

[5] H.J. Kim, T. Kim, N.J. Hoffman, D. Xiao, D.E. James, S.J. Humphrey, P. Yang, PhosR enables processing and functional analysis of phosphoproteomic data, Cell Rep, 34 (2021) 108771.

[6] H.J. Kim, T. Kim, D. Xiao, P. Yang, Protocol for the processing and downstream analysis of phosphoproteomic data with PhosR, STAR Protoc, 2 (2021) 100585.

[7] P.V. Hornbeck, B. Zhang, B. Murray, J.M. Kornhauser, V. Latham, E. Skrzypek, PhosphoSitePlus, 2014: mutations, PTMs and recalibrations, Nucleic Acids Res, 43 (2015) D512-520.

[8] P. Shannon, A. Markiel, O. Ozier, N.S. Baliga, J.T. Wang, D. Ramage, N. Amin, B. Schwikowski, T. Ideker, Cytoscape: a software environment for integrated models of biomolecular interaction networks, Genome Res, 13 (2003) 2498-2504.

[9] C.T. Rueden, J. Schindelin, M.C. Hiner, B.E. DeZonia, A.E. Walter, E.T. Arena, K.W. Eliceiri, ImageJ2: ImageJ for the next generation of scientific image data, BMC Bioinformatics, 18 (2017) 529.

[10] Y. Zhou, B. Zhou, L. Pache, M. Chang, A.H. Khodabakhshi, O. Tanaseichuk, C. Benner, S.K. Chanda, Metascape provides a biologist-oriented resource for the analysis of systems-level datasets, Nat Commun, 10 (2019) 1523.

[11] T. Li, J. Fu, Z. Zeng, D. Cohen, J. Li, Q. Chen, B. Li, X.S. Liu, TIMER2.0 for analysis of tumor-infiltrating immune cells, Nucleic Acids Res, 48 (2020) W509-W514.

[12] B. Li, E. Severson, J.C. Pignon, H. Zhao, T. Li, J. Novak, P. Jiang, H. Shen, J.C. Aster, S. Rodig, S. Signoretti, J.S. Liu, X.S. Liu, Comprehensive analyses of tumor immunity: implications for cancer immunotherapy, Genome Biol, 17 (2016) 174.

**Supplementary Figure Legends**

**Figure S1.** **Effects of PAKs knockout on cancer cell proliferation, apoptosis and death.** **a)** PAK1 and PAK4 protein expression were immunoblotted in KPC WT, PAK1 KO, PAK4 KO and PAK1&4 KO cell lines. **b)** Cell proliferation of KPC WT, PAK1 KO, PAK4 KO and PAK1&4 KO cell lines were measured by MTT at 4, 24, 48 and 72 hours. Cell proliferation measured at 4 hours of each cell line was taken as 100%. The apoptotic and dead cells were measured by Annexin-V analysis **(c, d)**. Statistical significance: *p<0.05, **p<0.01, ***p<0.001. All comparisons were made against WT unless otherwise indicated.

**Figure S2.** **Differential protein expression and pathways identified from global proteomic analysis.** **a)** Volcano plot of differentially expressed proteins between WT and PAK1 KO, PAK4 KO and PAK1&4 KO cancer cells respectively. **b)** Heatmap demonstrating expression profile of significant proteins grouped into four clusters by K means clustering. **c)** UMAP showed significant proteins with different colours indicating relevant clusters. **d-e)** Functional enrichment for KEGG and Reactome pathways indicated cell death and cell cycle-related pathways respectively.

**Figure S3.** **Mice body weight.** The body weight showed a steady increase in all mice for experiments described in Fig 1a (**a)**, Fig 1f **(b)**, Fig 2a **(c)** and Fig 3f **(d)**.

**Figure S4. Gating strategy for FACS of intra-tumoral lymphocyte infiltration in Figure 2 and Figure 4.**

**Figure S5.** **Effects of PAKs knockout on the tumour infiltration and activation of T cells by one-week tumour growth.** PAK4 KO increased the tumour infiltration and activation of CD8+ T cells **(a,b)** demonstrated by the increased Granzyme B (GrzB) positive and GrzB+ and perforin + CD8 T cells while PAK1KO decreased GrzB+ CD8 T cells and did not affect perforin+ and GrzB+ perforin+ CD8 T cells. PAK1&4KO increased the GrzB+ perforin+ CD8 T cells **(a,b)**. Similar results were obtained for CD4+ T cells in WT vs KO tumours **(c,d)**. PAK4 KO decreased regulatory T cells (Treg) and PAK1KO did not affect Treg **(e,f)**. PAK4KO increased PD1+ CD8+ T cells while PAK1KO decreased PD1+ CD8+ T cells **(g,h)**.Statistical significance: *p<0.05, **p<0.01, ***p<0.001. All comparisons were made against WT unless otherwise indicated.

**Figure S6. PAK4KO-stimulated immune response decreased from one to four weeks. a)** From the global proteomic analysis, the differences in the expression profile of significant proteins (grouped into four clusters by K means clustering and visualized by heatmap **(a)** and UMAP**(b)**) between WT and PAK4KO, were reduced from one week to four weeks, demonstrated in a heatmap **(a)**. **b)** UMAP showed significant proteins with different colours indicating relevant clusters. **c-d)** Immune related KEGG and Reactome pathway enrichment respectively. The phospho-proteomic analysis of KPC WT versus PAK4 KO tumours at one week showed differentially expressed phosphor-sites which were decreased by four weeks **(e)**. **f)** Kinase enrichment heatmap demonstrated top phospho-sites involved in kinase prediction. Kinase enrichment was based on significant phospho-sites as identified by ANOVA analysis between all four groups PAK4 KO-induced changes in kinase activation at one week **(g)** disappeared by four weeks **(h)**. Kinases with adjusted p-value <0.05 were highlighted in red.

**Figure S7.** **Effects of PAKs knockout on the tumour infiltration and activation of T cells by four-week tumour growth.** PAK4KO-induced reduction of infiltrating FoxP3 CD4 T cells at one week disappeared by four weeks **(a,b)** while PAK1KO increased FoxP3 CD4 T cells. Opposite to the PAK4KO-caused increase of PD1+ CD8+ T cells at one week, PAK4KO decreased PD1+ CD8+ T cells by four weeks **(c,d)**. Statistical significance: *p<0.05, **p<0.01, ***p<0.001. All comparisons were made against WT unless otherwise indicated.

**Figure S8. TCGA database analysis of 178 cases of pancreatic ductal adenocarcinoma.** **a-b)** Kaplan-Meier curves showed an inverse correlation between PAK1 and PAK4 gene expression and patient survival respectively. **c-d)** Top 200 PAK1 and PAK4 negatively correlated genes were identified and subjected to functional enrichment for GO, KEGG and Wikipath terms. The top 20 terms with the highest -log10(p-value) were summarized for PAK1 and PAK4 respectively. **e-f)** B-cell, CD4+ and CD8 + T cell infiltration levels were estimated by TIMER algorithm based on RNA sequencing data and correlated with PAK1 and PAK4 gene expression levels respectively.

**Supplementary Tables**

| Table S1. Buffer contents | |
| --- | --- |
| Buffer | Content |
| RIPA cell lysis buffer | 25mM Tris HCL, 150mM NaCl, 1% Triton X-100, 1% Na deoxycholate, 0.5% SDS, 1mM EGTA, PH 8 |
| SDS sample loading buffer | 125mM Tris, 20% Glycerol, 4% SDS, PH 6.8 |
| Digestion buffer | HBSS, 2% FBS, 1.25mg/ml collagenase IV |
| FACS buffer | 1X PBS, 2%FBS, 0.05% Sodium Azide |
| Tris-EDTA buffer | 10 mM Tris base, 1 mM EDTA solution, 0.05% Tween 20, PH 9.0 |

| Table S2. Primary antibodies for immunoblot and immunohistochemistry | | | |
| --- | --- | --- | --- |
| Protein target | Dilution | Cat. number | Company |
| PAK1 | Immunoblot 1:1000  Immunohistochemistry: 1:1000 | MA5-42513 | Invitrogen |
| PAK4 | Immunoblot: 1:1000  Immunohistochemistry: 1:500 | PA5-15120 | Invitrogen |
| CD4 | Immunohistochemistry 1:1000 | ab288724 | Abcam |
| CD8 | Immunohistochemistry: 1:2000 | ab237709 | Abcam |
| GAPDH | Immunoblot 1:10000 | 2118S | Cell Signaling & Technology |

| Table S3. Primary antibodies for flow cytometry | | | | |
| --- | --- | --- | --- | --- |
| Protein target | Fluorophore | Dilution | Cat. number | Company |
| CD45 | BV510 | 1:500 | 563891 | BD Biosciences |
| B220 | BV605 | 1:100 | 563708 | BD Biosciences |
| CD3 | PE | 1:100 | 553064 | BD Biosciences |
| CD4 | APC-Cy7 | 1:200 | 561830 | BD Biosciences |
| CD8a | PE-Cy7 | 1:200 | 561097 | BD Biosciences |
| PD1 | APC | 1:100 | 130-111-801 | Miltenyi Biotec |
| FoxP3 | BV421 | 1:200 | 562996 | BD Biosciences |
| Granzyme B | APC | 1:100 | 130-130-581 | Miltenyi Biotec |
| Perforin | Pacific Blue | 1:100 | 154407 | BioLegend |

| Table S4. Comparison of variables by PAK1 expression levels | | | | |
| --- | --- | --- | --- | --- |
| Variable | Low  (n=49) | High  (n=48) | t/ χ^2^/W | p-value |
| Age  (Mean (SD)) | 67.2 (10.8) | 64.7(10.4) | 1.150 | 0.253 |
| Sex  (Number (%))   - Male - Female | 20 (40.82%)  29 (50.18%) | 31 (64.58%)  17 (35.42%) | 4.581 | 0.032* |
| CD4  (Median (IQR) | 5.08 (5.69) | 5.41 (8.46) | 982 | 0.217 |
| CD8  (Median (IQR) | 2.76 (3.37) | 2.96 (3.01) | 1060 | 0.507 |
| Cancer Site  (Number (%))   - Head/neck - Body/tail - Multifocal | 41 (85.42%)  7 (14.58%)  0 | 43 (89.59%)  4 (8.33%)  1 (2.08%) | 1.866 | 0.393 |
| Resection Margin  (Number (%))   - R0 - R1 | 34 (69.39%)  15 (30.61%) | 27 (56.25%)  21 (43.75%) | 1.274 | 0.259 |
| Primary tumour size  (Mean (SD)) | 32.1 (9.45) | 35.1 (13.2) | -1.267 | 0.209 |
| T stage  (Number (%))   - 1 - 2 - 3 - 4 | 6 (12.24%)  35 (71.43%)  8 (16.33%)  0 | 6 (12.50%)  32 (66.67%)  9 (18.75%)  1 (2.08%) | 1.183 | 0.757 |
| N stage  (Number (%))   - 0 - 1 - 2 | 20 (40.82%)  19 (38.78%)  10 (20.41%) | 14 (29.17%)  16 (33.33%)  18 (37.50%) | 3.952 | 0.166 |
| M stage  (Number (%))   - 0 - 1 | 49 (100%)  0 (0%) | 47 (97.92%)  1 (2.08%) | 0.0001 | 0.992 |
| Grade  (Number (%))   - 1 - 2 - 3 | 3 (6.12%)  21 (42.86%)  25 (51.02%) | 0 (0%)  22 (46.81%)  25 (53.19%) | 2.9829 | 0.225 |
| Lymphovascular Invasion  (Number (%))   - Yes - No | 33 (68.75%)  15 (31.25%) | 33 (71.74%)  13 (28.26%) | 0.008 | 0.927 |
| Perineural Invasion  (Number (%))   - Yes - No | 14 (31.11%)  31 (68.89%) | 20 (43.48%)  26 (56.52%) | 1.005 | 0.316 |
| Adjuvant chemotherapy  (Number (%))   - Yes - No | 33 (73.33%)  12 (26.67%) | 42 (91.30%)  4 (8.70%) | 3.905 | 0.0481* |
| Death  (Number (%))   - No - Yes | 14 (28.57%)  35 (71.43%) | 10 (20.83%)  38 (79.17%) | 0.42 | 0.517 |
| Overall Survival in months  (Mean (SD)) | 38.3 (30.5) | 40.8 (34.3) | -0.373 | 0.710 |
| Abbreviations: SD: standard deviation; IQR: inter-quantile range; t: two-sided t test; χ^2^: Chi-square test; W: Mann-Whitney U test. Statistical significance: p<0.05^*^, p<0.01^**^, p<0.001^***^. | | | | |

| Table S5. Comparison of variables by PAK4 expression levels | | | | |
| --- | --- | --- | --- | --- |
| Variable | Low  (n=47) | High  (n=48) | t/ χ^2^/W | p-value |
| Age  (Mean (SD)) | 66.2 (11.5) | 65.8 (9.99) | 0.162 | 0.872 |
| Sex  (Number (%))   - Male - Female | 24 (51.06%)  23 (48.94%) | 27 (56.25%)  21 (43.75%) | 0.091 | 0.763 |
| CD4  (Median (IQR)) | 4.86 (6.05) | 8.88 (7.35) | 738 | 0.003** |
| CD8  (Median (IQR)) | 3.30 (3.39) | 3.71 (3.11) | 966 | 0.231 |
| Cancer Site  (Number (%))   - Head/neck - Body/tail - Multifocal | 41 (89.13%)  5 (10.87%)  0 | 41 (85.42%)  6 (12.5%)  1 (2.08%) | 1.049 | 0.592 |
| Resection Margin  (Number (%))   - R0 - R1 | 30 (63.83%)  17 (36.17%) | 30 (62.50%)  18 (37.50%) | 0 | 1 |
| Primary tumour size  (Mean (SD)) | 33.3 (12.5) | 34.1 (10.7) | -0.337 | 0.737 |
| T stage  (Number (%))   - 1 - 2 - 3 - 4 | 7 (14.89%)  33 (70.21%)  7 (14.89%)  0 (0%) | 5 (10.42%)  32 (66.67%)  10 (20.83%)  1 (2.08%) | 1.868 | 0.600 |
| N stage  (Number (%))   - 0 - 1 - 2 | 16 (34.04%)  17 (36.17%)  14 (29.79%) | 18 (37.5%)  17 (35.42%)  13 (27.08%) | 0.144 | 0.931 |
| M stage  (Number (%))   - 0 - 1 | 46 (97.87%)  1 (2.13%) | 48 (100%)  0 | 0.0001 | 0.992 |
| Grade  (Number (%))   - 1 - 2 - 3 | 2 (4.35%)  17 (36.96%)  27 (58.70%) | 1 (2.08%)  26 (54.17%)  21 (43.75%) | 2.926 | 0.232 |
| Lymphovascular Invasion  (Number (%))   - Yes - No | 36  10 | 29  17 | 1.887 | 0.170 |
| Perineural Invasion  (Number (%))   - Yes - No | 17 (38.64%)  27 (61.36%) | 16 (35.56%)  29 (64.44%) | 0.007 | 0.935 |
| Adjuvant chemotherapy  (Number (%))   - Yes - No | 36 (80%)  9 (20%) | 37 (84.09%)  7 (15.91%) | 0.051 | 0.821 |
| Death  (Number (%))   - No - Yes | 15 (31.91%)  32 (68.09%) | 9 (18.75%)  39 (81.25%) | 1.538 | 0.215 |
| Overall Survival in months  (Mean (SD)) | 38.8 (35) | 41.1 (30) | -0.352 | 0.726 |
| Abbreviations: SD: standard deviation; IQR: inter-quantile range; t: two-sided t test; χ^2^: Chi-square test; W: Mann-Whitney U test. Statistical significance: p<0.05^*^, p<0.01^**^, p<0.001^***^. | | | | |

| Table S6. Univariate and multivariate linear regression for CD4 T cell level | | | | |
| --- | --- | --- | --- | --- |
|  | Univariate | | Multivariate | |
| Variable | Correlation Coefficient (r) | p-value | Correlation Coefficient (r) | p-value |
| Age | -0.006 | 0.924 | -0.063 | 0.231 |
| Sex   - Male - Female | -  -1.789 | -  0.189 | -  0.623 | -  0.590 |
| PAK1 | 19.735 | 0.002** | 14.574 | 0.315 |
| PAK4 | 64.715 | <0.001*** | 9.320 | 0.720 |
| PAK1:PAK4  Interaction | - | - | 6.250 | 0.936 |
| CD8 | 1.3918 | <0.001*** | 1.157 | <0.001*** |
| Cancer Site   - Head/neck - Body/tail - Multifocal | -  0.885  6.26 | -  0.682  0.356 | -  -1.424  1.097 | -  0.513  0.823 |
| Resection Margin   - R0 - R1 | -  -0.115 | -  0.935 | -  -1.727 | -  0.208 |
| T stage   - 1 - 2 - 3 - 4 | -  -0.743  -0.065  -1.286 | -  0.726  0.98  0.855 | -  1.239  1.276  3.385 | -  0.466  0.499  0.481 |
| N stage   - 0 - 1 - 2 | -  1.235  -0.378 | -  0.447  0.825 | -  2.073  0.001 | -  0.149  1 |
| M stage   - 0 - 1 | -  2.823 | -  0.675 | -  1.814 | -  0.707 |
| Grade   - 1 - 2 - 3 | -  -4.804  -5.437 | -  0.231  0.174 | -  -2.435  -3.055 | -  0.496  0.383 |
| Lymphovascular Invasion   - Yes - No | -  2.271 | -  0.14 | -  3.190 | -  0.015* |
| Perineural Invasion   - Yes - No | -  0.058 | -  0.969 | -  -1.734 | -  0.164 |
| Adjuvant chemotherapy   - Yes - No | -  2.068 | -  0.172 | -  3.209 | -  0.936 |
| Statistical significance: p<0.05^*^, p<0.01^**^, p<0.001^***^. Statistical significance: p<0.05^*^, p<0.01^**^, p<0.001^***^. | | | | |

| Table S7. Univariate and multivariate linear regression for CD8 T cell level | | | | |
| --- | --- | --- | --- | --- |
|  | Univariate | | Multivariate | |
| Variable | Correlation Coefficient (r) | p-value | Correlation Coefficient(r) | p-value |
| Age | 0.003 | 0.918 | 0.005 | 0.840 |
| Sex   - Male - Female | -  -0.790 | -  0.136 | -  -0.510 | -  0.321 |
| PAK1 | 2.358 | 0.355 | -5.476 | 0.399 |
| PAK4 | 11.07 | 0.045* | -5.314 | 0.647 |
| PAK1:PAK4  Interaction | - | - | 26.070 | 0.455 |
| CD4 | 0.215 | <0.001*** | 0.230 | <0.001*** |
| Cancer Site   - Head/neck - Body/tail - Multifocal | -  0.546  1.714 | -  0.52  0.52 | -  -0.522  1.286 | -  0.591  0.556 |
| Resection Margin   - R0 - R1 | -  0.768 | -  0.162 | -  0.635 | -  0.301 |
| T stage   - 1 - 2 - 3 - 4 | -  -0.699  -0.039  -2.093 | -  0.369  0.967  0.444 | -  -1.366  -0.923  -2.019 | -  0.068  0.271  0.345 |
| N stage   - 0 - 1 - 2 | -  -1.739  -1.263 | -  0.005**  0.051 | -  -2.052  -0.980 | -  <0.001***  0.129 |
| M stage   - 0 - 1 | -  -0.041 | -  0.988 | -  -1.157 | -  0.591 |
| Grade   - 1 - 2 - 3 | -  -1.280  -1.102 | -  0.420  0.485 | -  -0.927  -0.466 | -  0.562  0.766 |
| Lymphovascular Invasion   - Yes - No | -  -0.007 | -  0.99 | -  -0.928 | -  0.120 |
| Perineural Invasion   - Yes - No | -  -0.043 | -  0.94 | -  -0.033 | -  0.952 |
| Adjuvant chemotherapy   - Yes - No | -  0.748 | -  0.237 | -  -0.020 | -  0.979 |
| Statistical significance: p<0.05^*^, p<0.01^**^, p<0.001^***^. Statistical significance: p<0.05^*^, p<0.01^**^, p<0.001^***^. | | | | |
