# Supplementary material for: Inhibition of P21-activated kinases 1 and 4 synergistically suppresses the growth of pancreatic cancer by stimulating anti-tumour immunity
Source: Cell Commun Signal. 2024 May 27;22:287. doi: 10.1186/s12964-024-01670-2 (PMC11129409; doi:10.1186/s12964-024-01670-2)
Supplement: Supplementary file 2 — Supplementary Material 2. [file 12964_2024_1670_MOESM2_ESM.pdf]

Figure S1

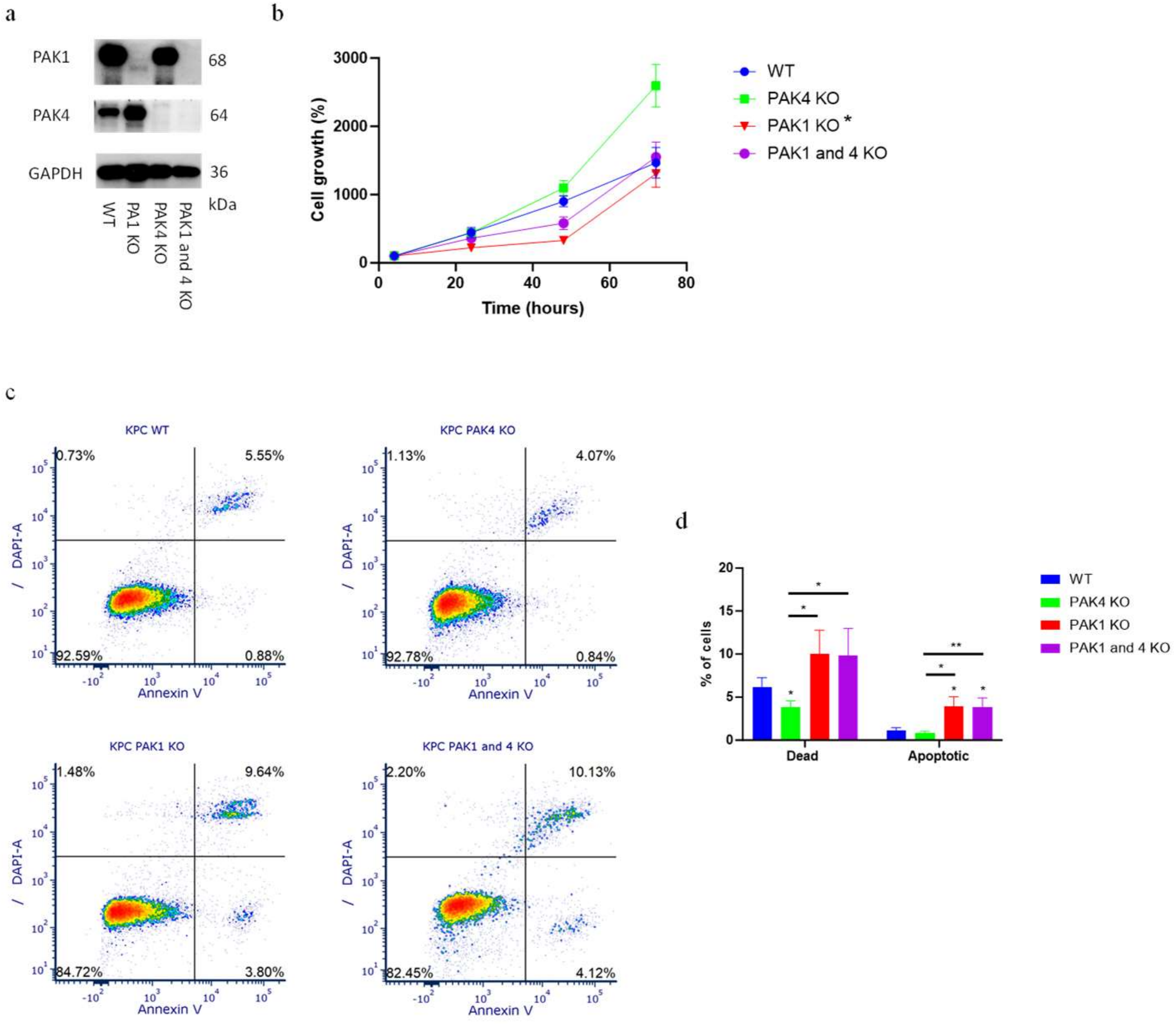
a
PAK1
68
PAK4
64
GAPDH
36
kDa
WT
PA1 KO
PAK4 KO
PAK1 and 4 KO
b
Cell growth (%)
Time (hours)
WT
PAK4 KO
PAK1 KO *
PAK1 and 4 KO
c
KPC WT
0.73%
5.55%
92.59%
0.88%
KPC PAK4 KO
1.13%
4.07%
92.78%
0.84%
KPC PAK1 KO
1.48%
9.64%
84.72%
3.80%
KPC PAK1 and 4 KO
2.20%
10.13%
82.45%
4.12%
DAPI-A
Annexin V
d
% of cells
Dead
Apoptotic
WT
PAK4 KO
PAK1 KO
PAK1 and 4 KO

Figure S2

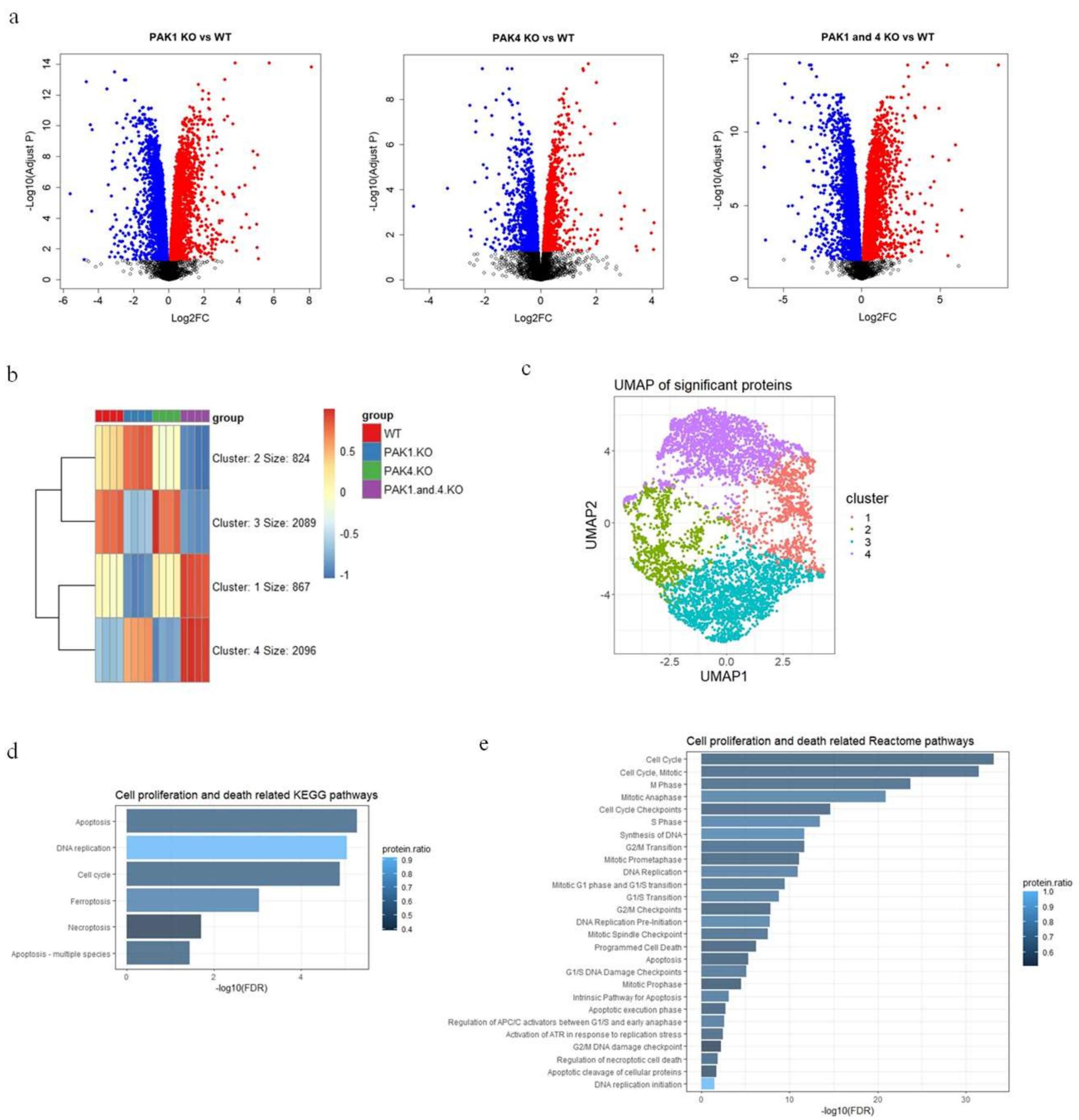

a
PAK1 KO vs WT
PAK4 KO vs WT
PAK1 and 4 KO vs WT
-Log10(Adjust P)
Log2FC
b
group
Cluster: 2 Size: 824
Cluster: 3 Size: 2089
Cluster: 1 Size: 867
Cluster: 4 Size: 2096
WT
PAK1.KO
PAK4.KO
PAK1.and.4.KO
c
UMAP of significant proteins
UMAP1
UMAP2
cluster
d
Cell proliferation and death related KEGG pathways
Apoptosis
DNA replication
Cell cycle
Ferroptosis
Necroptosis
Apoptosis - multiple species
protein.ratio
-log10(FDR)
e
Cell proliferation and death related Reactome pathways
Cell Cycle
Cell Cycle, Mitotic
M Phase
Mitotic Anaphase
Cell Cycle Checkpoints
S Phase
Synthesis of DNA
G2/M Transition
Mitotic Prometaphase
DNA Replication
Mitotic G1 phase and G1/S transition
G1/S Transition
G2/M Checkpoints
DNA Replication Pre-Initiation
Mitotic Spindle Checkpoint
Programmed Cell Death
Apoptosis
G1/S DNA Damage Checkpoints
Mitotic Prophase
Intrinsic Pathway for Apoptosis
Apoptotic execution phase
Regulation of APC/C activators between G1/S and early anaphase
Activation of ATR in response to replication stress
G2/M DNA damage checkpoint
Regulation of necroptotic cell death
Apoptotic cleavage of cellular proteins
DNA replication initiation
protein.ratio
-log10(FDR)

Figure S3

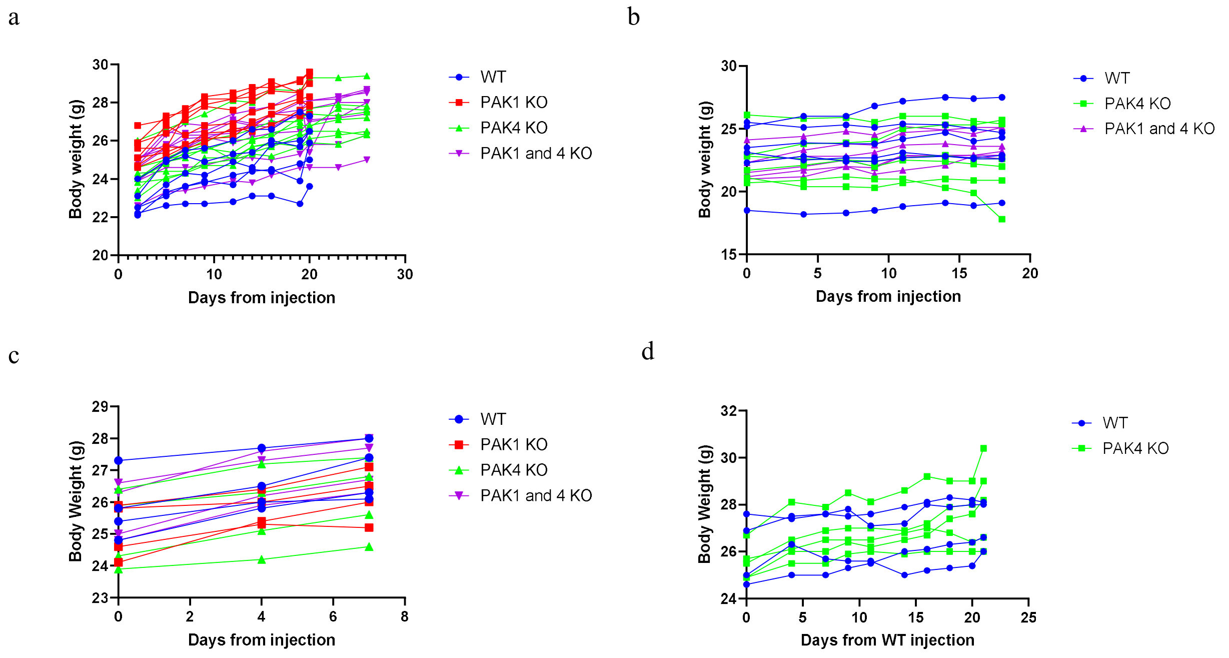

a
Body weight (g)
Days from injection
WT
PAK1 KO
PAK4 KO
PAK1 and 4 KO
b
Body weight (g)
Days from injection
WT
PAK4 KO
PAK1 and 4 KO
c
Body Weight (g)
Days from injection
WT
PAK1 KO
PAK4 KO
PAK1 and 4 KO
d
Body Weight (g)
Days from WT injection
WT
PAK4 KO

Figure S4

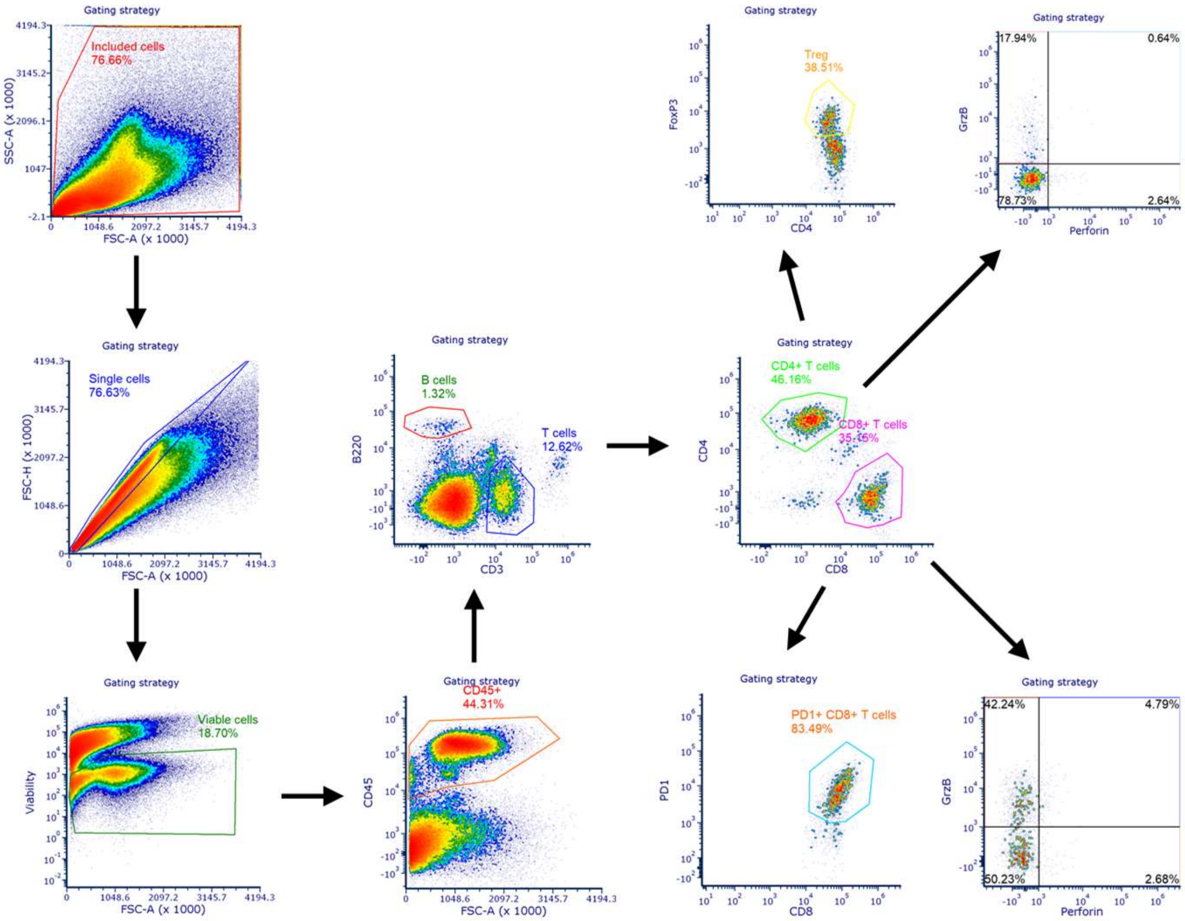

Gating strategy
Included cells
76.66%
SSC-A (x 1000)
FSC-A (x 1000)
Single cells
76.63%
FSC-H (x 1000)
Viable cells
18.70%
Viability
CD45+
44.31%
CD45
B cells
1.32%
T cells
12.62%
B220
CD3
CD4+ T cells
46.16%
CD8+ T cells
CD4
CD8
Treg
38.51%
FoxP3
17.94%
0.64%
78.73%
2.64%
GrzB
Perforin
PD1+ CD8+ T cells
83.49%
PD1
42.24%
4.79%
50.23%
2.68%

Figure S5

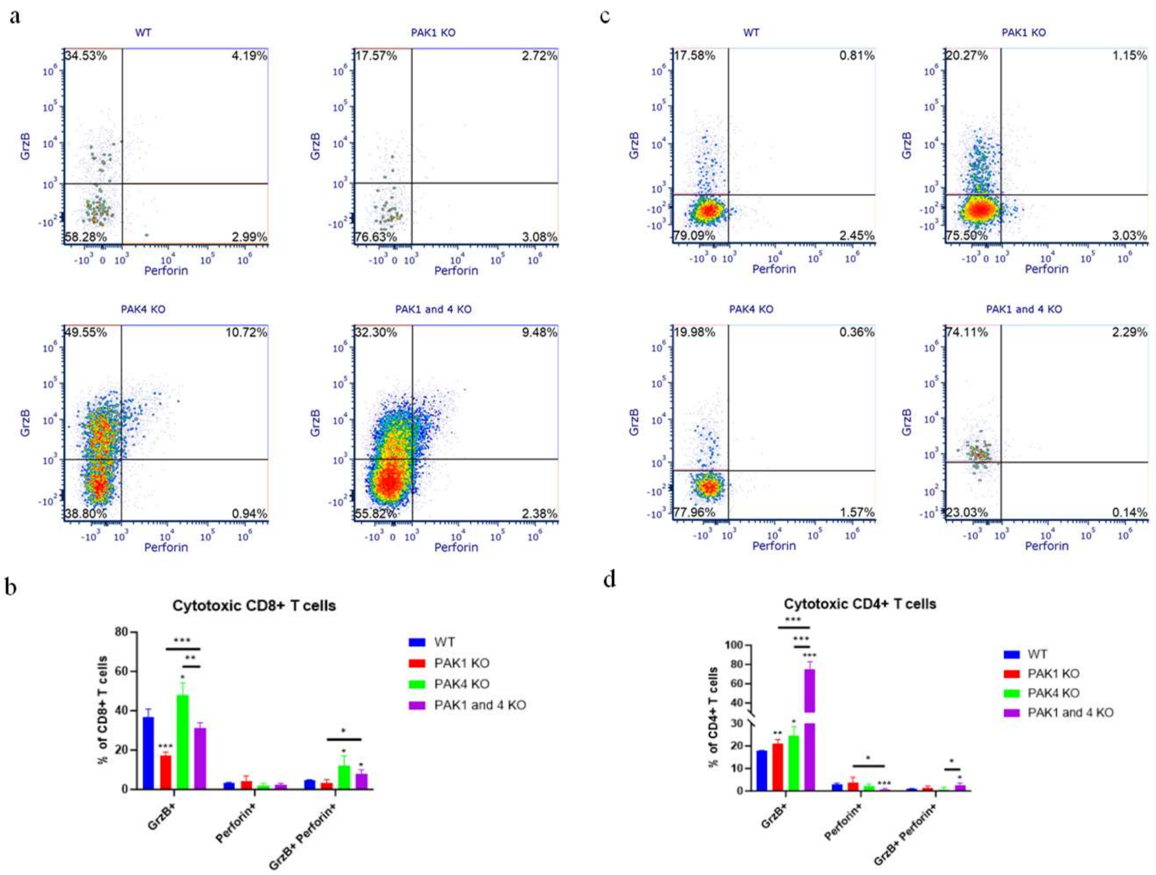
a
WT
PAK1 KO
PAK4 KO
PAK1 and 4 KO
GrzB
Perforin
c
b
Cytotoxic CD8+ T cells
% of CD8+ T cells
GrzB+
Perforin+
GrzB+ Perforin+
WT
PAK1 KO
PAK4 KO
PAK1 and 4 KO
d
Cytotoxic CD4+ T cells
% of CD4+ T cells

Figure S5 (cont.)

e

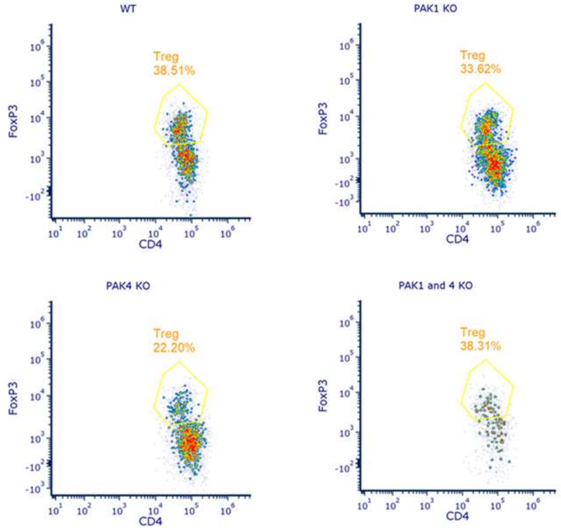

f

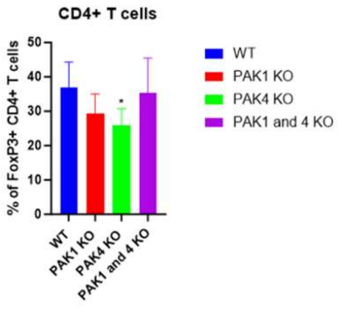

g

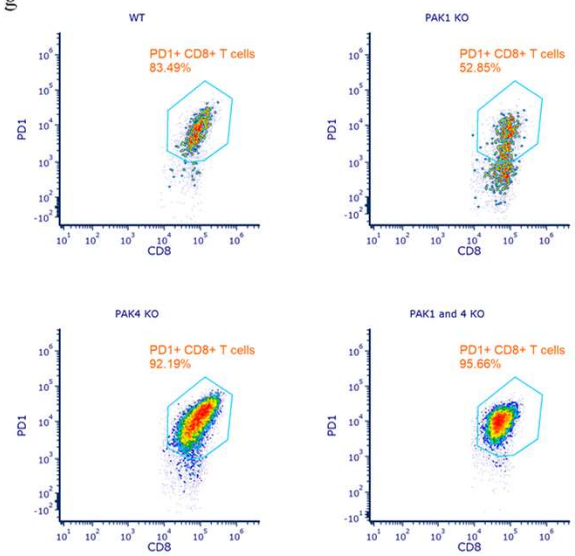

h

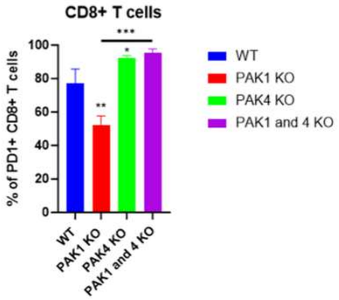

Figure S6

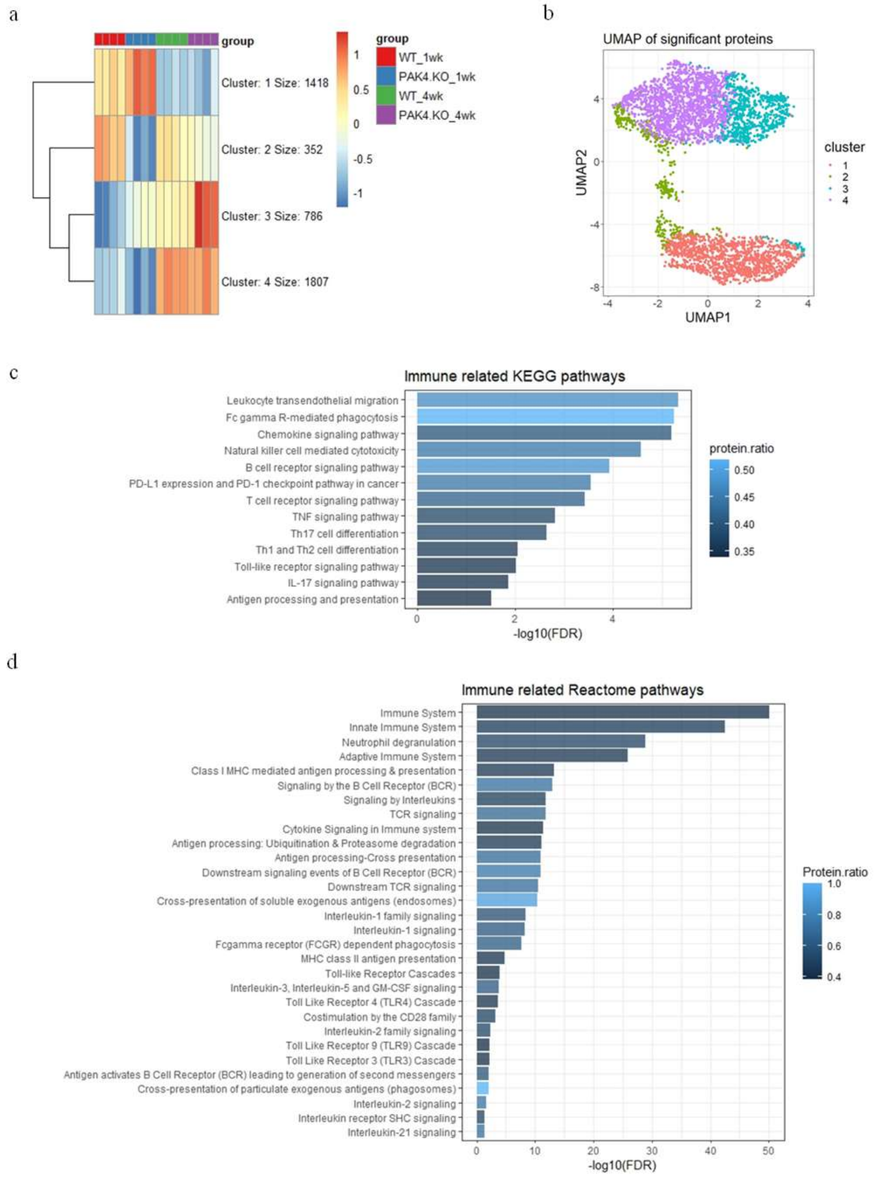
a
group
Cluster: 1 Size: 1418
Cluster: 2 Size: 352
Cluster: 3 Size: 786
Cluster: 4 Size: 1807
1
0.5
0
-0.5
-1
group
WT_1wk
PAK4.KO_1wk
WT_4wk
PAK4.KO_4wk
b
UMAP of significant proteins
UMAP2
UMAP1
cluster
1
2
3
4
c
Immune related KEGG pathways
Leukocyte transendothelial migration
Fc gamma R-mediated phagocytosis
Chemokine signaling pathway
Natural killer cell mediated cytotoxicity
B cell receptor signaling pathway
PD-L1 expression and PD-1 checkpoint pathway in cancer
T cell receptor signaling pathway
TNF signaling pathway
Th17 cell differentiation
Th1 and Th2 cell differentiation
Toll-like receptor signaling pathway
IL-17 signaling pathway
Antigen processing and presentation
-log10(FDR)
protein.ratio
0.50
0.45
0.40
0.35
d
Immune related Reactome pathways
Immune System
Innate Immune System
Neutrophil degranulation
Adaptive Immune System
Class I MHC mediated antigen processing & presentation
Signaling by the B Cell Receptor (BCR)
Signaling by Interleukins
TCR signaling
Cytokine Signaling in Immune system
Antigen processing: Ubiquitination & Proteasome degradation
Antigen processing-Cross presentation
Downstream signaling events of B Cell Receptor (BCR)
Downstream TCR signaling
Cross-presentation of soluble exogenous antigens (endosomes)
Interleukin-1 family signaling
Interleukin-1 signaling
Fcgamma receptor (FCGR) dependent phagocytosis
MHC class II antigen presentation
Toll-like Receptor Cascades
Interleukin-3, Interleukin-5 and GM-CSF signaling
Toll Like Receptor 4 (TLR4) Cascade
Costimulation by the CD28 family
Interleukin-2 family signaling
Toll Like Receptor 9 (TLR9) Cascade
Toll Like Receptor 3 (TLR3) Cascade
Antigen activates B Cell Receptor (BCR) leading to generation of second messengers
Cross-presentation of particulate exogenous antigens (phagosomes)
Interleukin-2 signaling
Interleukin receptor SHC signaling
Interleukin-21 signaling
-log10(FDR)
Protein.ratio
1.0
0.8
0.6
0.4

Figure S6 (cont)

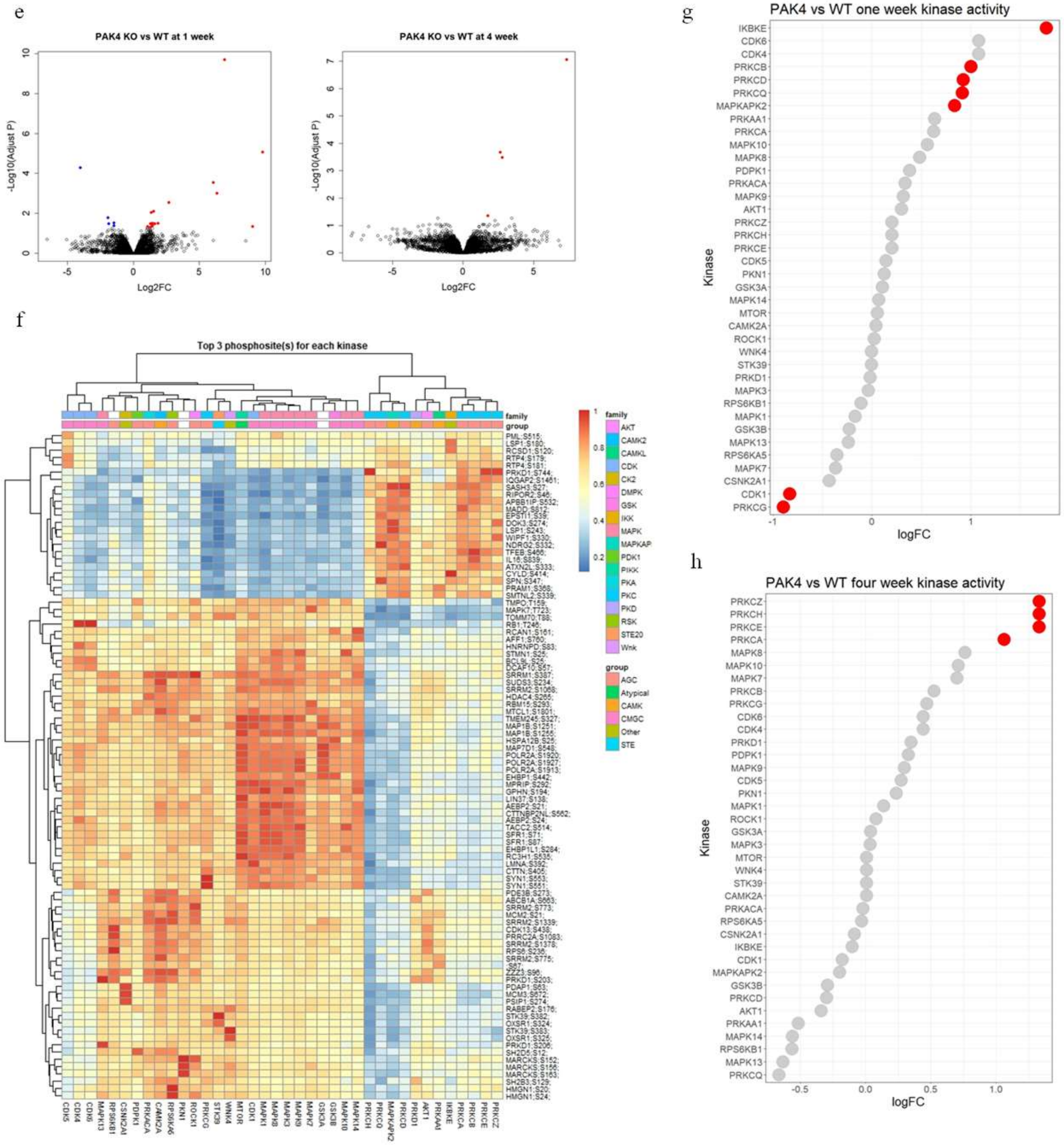

Figure S7

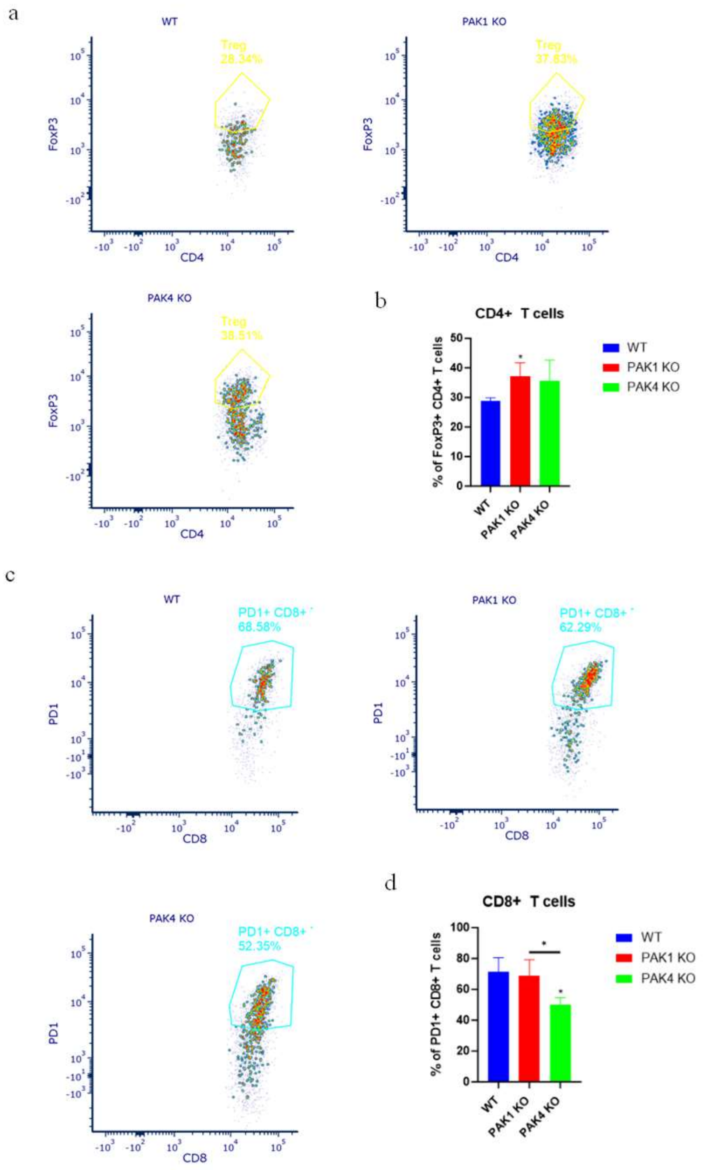
a
WT
Treg
28.34%
PAK1 KO
Treg
37.83%
PAK4 KO
Treg
38.51%
FoxP3
CD4
b
CD4+ T cells
% of FoxP3+ CD4+ T cells
WT
PAK1 KO
PAK4 KO
c
WT
PD1+ CD8+
68.58%
PAK1 KO
PD1+ CD8+
62.29%
PAK4 KO
PD1+ CD8+
52.35%
PD1
CD8
d
CD8+ T cells
% of PD1+ CD8+ T cells
WT
PAK1 KO
PAK4 KO

Figure S8

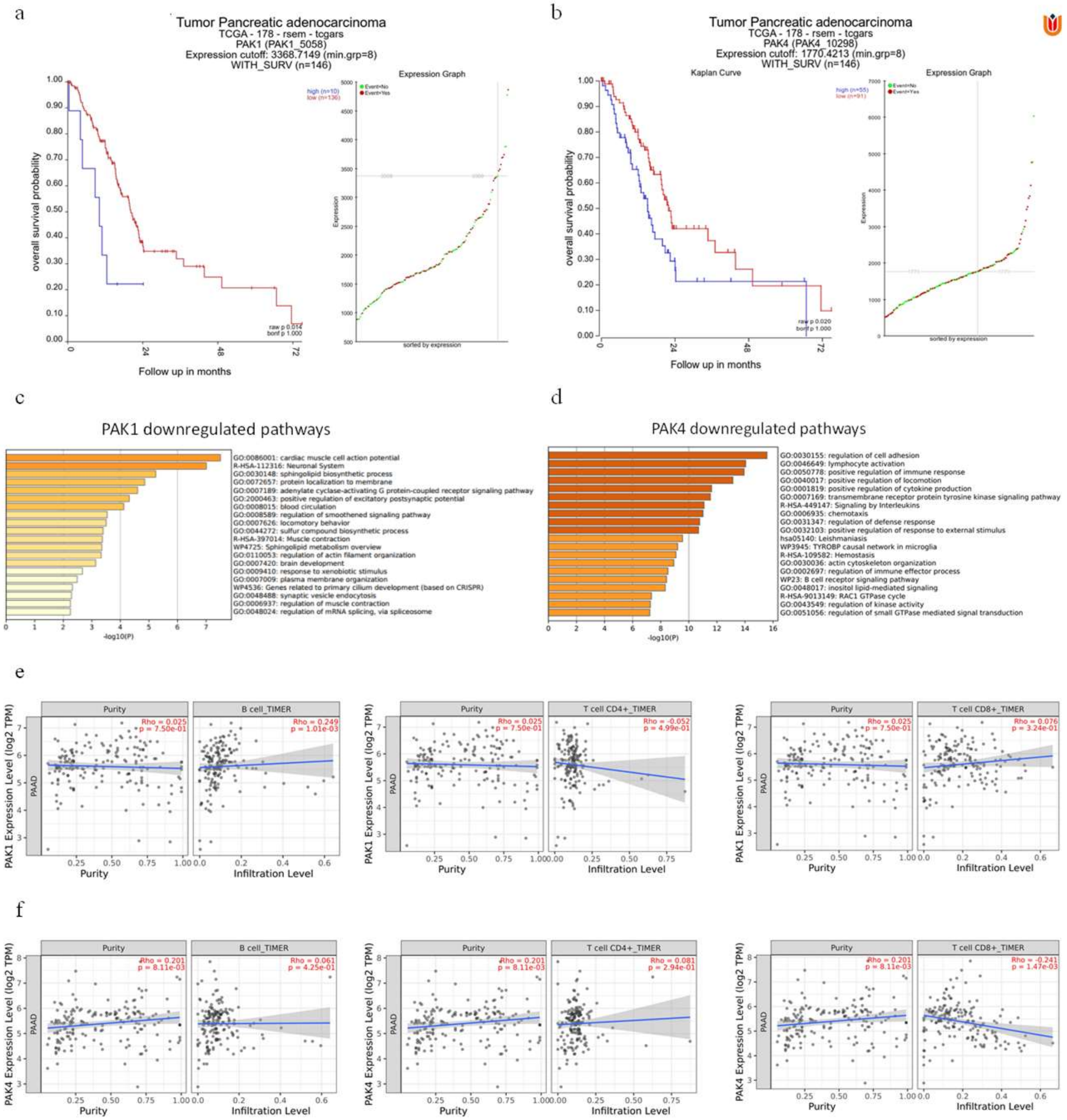
a
Tumor Pancreatic adenocarcinoma
TCGA - 178 - rsem - tcgars
PAK1 (PAK1_5058)
Expression cutoff: 3368.7149 (min.grp=8)
WITH_SURV (n=146)
overall survival probability
Follow up in months
raw p 0.014
bonf p 1.000
Expression Graph
Event=No
Event=Yes
Expression
sorted by expression
b
Tumor Pancreatic adenocarcinoma
TCGA - 178 - rsem - tcgars
PAK4 (PAK4_10298)
Expression cutoff: 1770.4213 (min.grp=8)
WITH_SURV (n=146)
Kaplan Curve
overall survival probability
Follow up in months
raw p 0.020
bonf p 1.000
Expression Graph
Event=No
Event=Yes
Expression
sorted by expression
c
PAK1 downregulated pathways
GO:0086001: cardiac muscle cell action potential
R-HSA-112316: Neuronal System
GO:0030148: sphingolipid biosynthetic process
GO:0072657: protein localization to membrane
GO:0007189: adenylate cyclase-activating G protein-coupled receptor signaling pathway
GO:2000463: positive regulation of excitatory postsynaptic potential
GO:0008015: blood circulation
GO:0008589: regulation of smoothened signaling pathway
GO:0007626: locomotory behavior
GO:0044272: sulfur compound biosynthetic process
R-HSA-397014: Muscle contraction
WP4725: Sphingolipid metabolism overview
GO:0110053: regulation of actin filament organization
GO:0007420: brain development
GO:0009410: response to xenobiotic stimulus
GO:0007009: plasma membrane organization
WP4536: Genes related to primary cilium development (based on CRISPR)
GO:0048488: synaptic vesicle endocytosis
GO:0006937: regulation of muscle contraction
GO:0048024: regulation of mRNA splicing, via spliceosome
-log10(P)
d
PAK4 downregulated pathways
GO:0030155: regulation of cell adhesion
GO:0046649: lymphocyte activation
GO:0050778: positive regulation of immune response
GO:0040017: positive regulation of locomotion
GO:0001819: positive regulation of cytokine production
GO:0007169: transmembrane receptor protein tyrosine kinase signaling pathway
R-HSA-449147: Signaling by Interleukins
GO:0006935: chemotaxis
GO:0031347: regulation of defense response
GO:0032103: positive regulation of response to external stimulus
hsa05140: Leishmaniasis
WP3945: TYROBP causal network in microglia
R-HSA-109582: Hemostasis
GO:0030036: actin cytoskeleton organization
GO:0002697: regulation of immune effector process
WP23: B cell receptor signaling pathway
GO:0048017: inositol lipid-mediated signaling
R-HSA-9013149: RAC1 GTPase cycle
GO:0043549: regulation of kinase activity
GO:0051056: regulation of small GTPase mediated signal transduction
-log10(P)
e
Purity
B cell_TIMER
Rho = 0.025
p = 7.50e-01
Rho = 0.249
p = 1.01e-03
PAK1 Expression Level (log2 TPM)
PAAD
Purity
Infiltration Level
T cell CD4+_TIMER
Rho = -0.052
p = 4.99e-01
T cell CD8+_TIMER
Rho = 0.076
p = 3.24e-01
f
Purity
B cell_TIMER
Rho = 0.201
p = 8.11e-03
Rho = 0.061
p = 4.25e-01
PAK4 Expression Level (log2 TPM)
PAAD
Purity
Infiltration Level
T cell CD4+_TIMER
Rho = 0.081
p = 2.94e-01
T cell CD8+_TIMER
Rho = -0.241
p = 1.47e-03
