# Supplementary material for: Inhibition of P21-activated kinases 1 and 4 synergistically suppresses the growth of pancreatic cancer by stimulating anti-tumour immunity
Source: Cell Commun Signal. 2024 May 27;22:287. doi: 10.1186/s12964-024-01670-2 (PMC11129409; doi:10.1186/s12964-024-01670-2)
Supplement: Supplementary file 3 — Supplementary Material 3. [file 12964_2024_1670_MOESM3_ESM.pptx]

## Slide 1
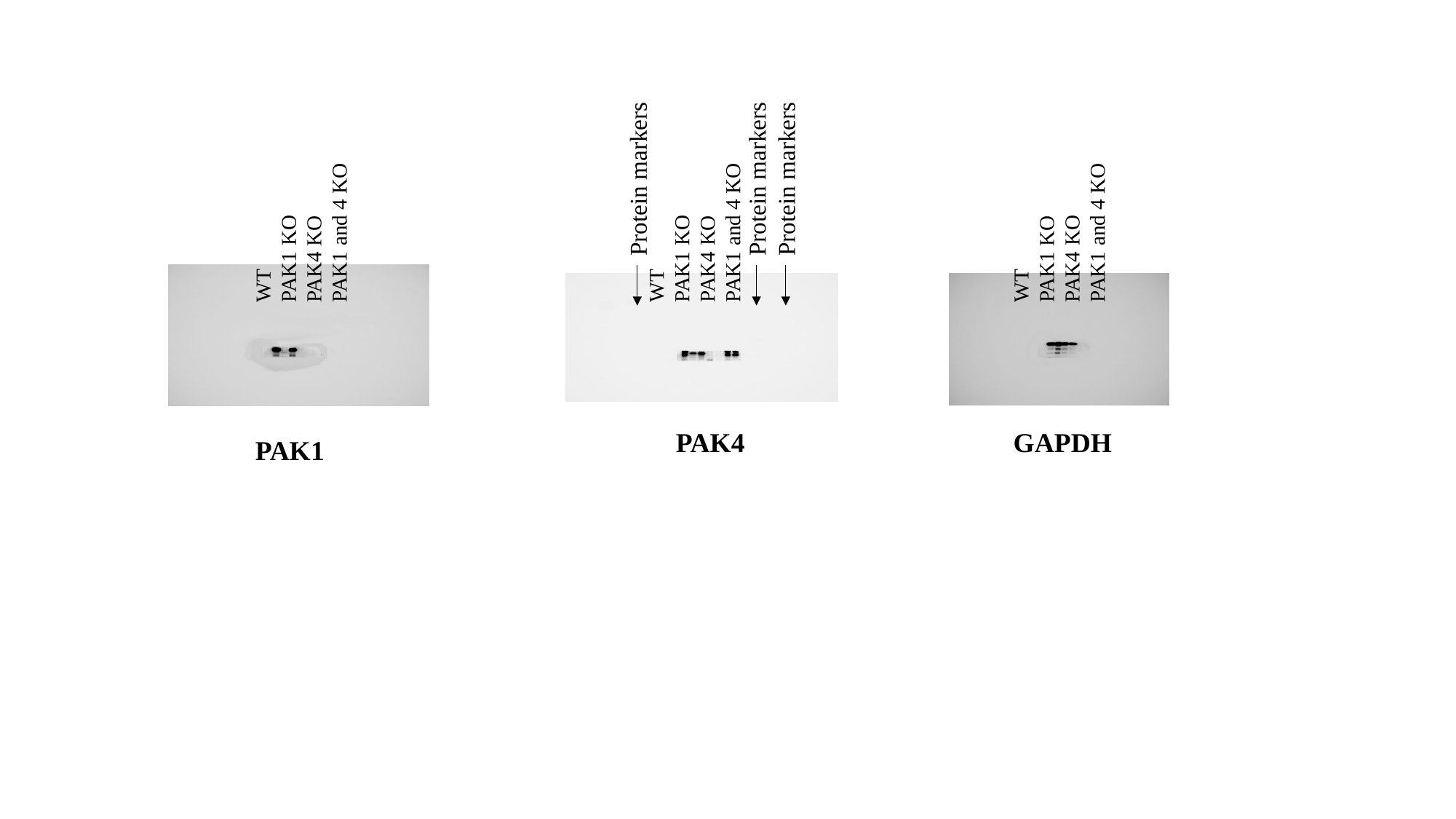

Protein markers
Protein markers
Protein markers
WT
PAK1 KO
PAK4 KO
PAK1 and 4 KO
WT
PAK1 KO
PAK4 KO
PAK1 and 4 KO
WT
PAK1 KO
PAK4 KO
PAK1 and 4 KO
PAK4
GAPDH
PAK1
